# Monovalent, bivalent and biparatopic nanobodies targeting S1 protein of porcine epidemic diarrhea virus efficiently neutralized the virus infectivity

**DOI:** 10.1186/s12917-024-04151-3

**Published:** 2024-07-30

**Authors:** Huai-rui Qin, Zhi Cao, Feng-zhe Lu, Wei Wang, Wenhui Zhao, Guimei Li, Hongliang Zhang, Shubai Wang, Zhihua Qin

**Affiliations:** College of Veterinary Medicine, Qingdao Agricultural University, Qingdao, 266109 China

**Keywords:** PEDV, S1 protein, Nanobody, Multivalent nanobodies, Antiviral effect

## Abstract

**Background:**

Porcine epidemic diarrhea virus (PEDV) is a highly contagious coronavirus that causes severe diarrhea and death in neonatal piglets, which has brought huge economic losses to the pork industry worldwide since its first discovery in the early 1970s in Europe. Passive immunization with neutralizing antibodies against PEDV is an effective prevention measure. To date, there are no effective therapeutic drugs to treat the PEDV infection.

**Results:**

We conducted a screening of specific nanobodies against the S1 protein from a phage display library obtained from immunized alpacas. Through competitive binding to antigenic epitopes, we selected instead of chose nanobodies with high affinity and constructed a multivalent tandem. These nanobodies were shown to inhibit PEDV infectivity by the neutralization assay. The antiviral capacity of nanobody was found to display a dose-dependent pattern, as demonstrated by IFA, TCID_50_, and qRT-PCR analyses. Notably, biparatopic nanobody SF-B exhibited superior antiviral activity. Nanobodies exhibited low cytotoxicity and high stability even under harsh temperature and pH conditions, demonstrating their potential practical applicability to animals.

**Conclusions:**

Nanobodies exhibit remarkable biological properties and antiviral effects, rendering them a promising candidate for the development of anti-PEDV drugs.

**Supplementary Information:**

The online version contains supplementary material available at 10.1186/s12917-024-04151-3.

## Introduction

Porcine epidemic diarrhea (PED) is an acute, highly contagious enteric disease in pigs caused by porcine epidemic diarrhea virus (PEDV) [1]. The characteristic clinical signs of PEDV infection are enteritis, vomiting, watery diarrhea, and dehydration [2]. Since its discovery in the early 1970s in Europe, the disease has spread globally, having brought huge economic losses to the pork industry worldwide [3]. In 2010, a variant of highly pathogenic G2 PEDV emerged in southern China which increased the morbidity and mortality of PED to 80–100%, resulting in the death of more than 1 million piglets [4]. Later, in April 2013, the outbreak of PED in the United States and neighboring countries (Canada and Mexico) caused the death of > 8 million piglets in the US alone [5]. PEDV infection of older pigs results in considerably lower morbidity and mortality, but before infection can still result in decreased growth performance, as well as posing a high risk of PEDV infection to their offspring [6]. At present, PED remains a major concern for the pig industry, due to the continuous emergence of recombinant mutant PEDV strains, the poor clinical protection of commercial vaccines, and the lack of effective antiviral drugs and treatment strategies [7].

The PEDV S protein is a 1386 residue glycoprotein of 180–220 kilodaltons in size [8]. Trimers of this S protein form the club-shaped, ± 20 nm long spikes on the virion surface that provide the coronavirus its typical crown-like appearance on electron micrographs [9]. Like other CoV spike proteins, the S protein is a type I glycoprotein that plays a crucial role in virus attachment, receptor binding, cell membrane fusion, entry and induction of neutralizing antibodies [10]. The S protein can be cleaved by host protease into S1 (residues 1-789) and S2 subunits (residues 790–1386) [11]. The S1 subunit contains the N-terminal domain (NTD, residues 1-233) that shows sialic acid binding activity and the C-terminal domain (CTD, residues 253–638) that attaches to the cell surface receptor [12]. The S2 subunit mediates virus-cell membrane fusion [13]. Several neutralizing epitopes have been identified on the S protein sequence that induces protective immunity against PEDV and are major target for neutralizing antibody production, subunit vaccine studies, and antiviral drugs [14]. Immunization of pregnant sows with the PEDV S1 protein could provide passive immunity against PEDV to suckling piglets through colostrum and milk [15]. In addition to that, egg yolk antibodies (IgY) against the S1 domain of the spike protein effectively protect neonatal piglets against PEDV [16].

With advances in biotechnology, genetically engineered recombinant antibody fragments are increasingly being used in medical diagnosis and therapy in many diseases. VHH are also known as nanobodies, which are immune fragments derived from the unique heavy-chain-only antibodies found in camelid species such as alpacas [17]. Compared with traditional antibodies, nanobodies (Nbs), which are the smallest antibodies with complete antigen-binding sites, have the advantages of small size, high affinity, high specificity and stability, good solubility, low immunogenicity, and strong penetrating ability [18]. Moreover, because the molecular weights of nanobodies are only approximately 15 kDa and they are associated with concave epitopes, nanobodies might be better adapted to access hidden targets and cryptic sites than antibodies [19]. Nanobodies typically have high yields and lower costs during recombinant production and allow straightforward protein engineering, for example fusing Fc domains or building multivalent nanobodies [20]. Multivalent presentations increase the binding avidity to the molecular target and thus the biological potency of such compounds [21]. A study has demonstrated that nanobodies targeting human host viruses or animal host viruses can inhibit the replication and proliferation of these viruses in host cells, showing favorable antiviral activity [22]. Thus, nanobodies are considered a potential reagent for the prevention and treatment of viral diseases.

At present, there have been no reports of the selection of neutralizing nanobodies against PEDV. In this study, we constructed a phage display library of nanobodies using peripheral blood lymphocytes from alpaca immunized with PEDV S1 protein. Three specific nanobodies against PEDV S1 protein were selected through phage display technology and their neutralization efficiency was evaluated. Based on the above results, bivalent and biparatopic nanobodies were constructed and the effect of multivalent nanobodies on PEDV replication was evaluated in vitro. The results indicated that multivalent nanobodies had better antiviral bioefficacy than monovalent nanobodies within the safe concentration range used. Our results provide a foundation for the development of nanobody-based drugs for the prevention and treatment of PEDV infection.

## Results

### Construction of the VHH library

**Fig. 1:**
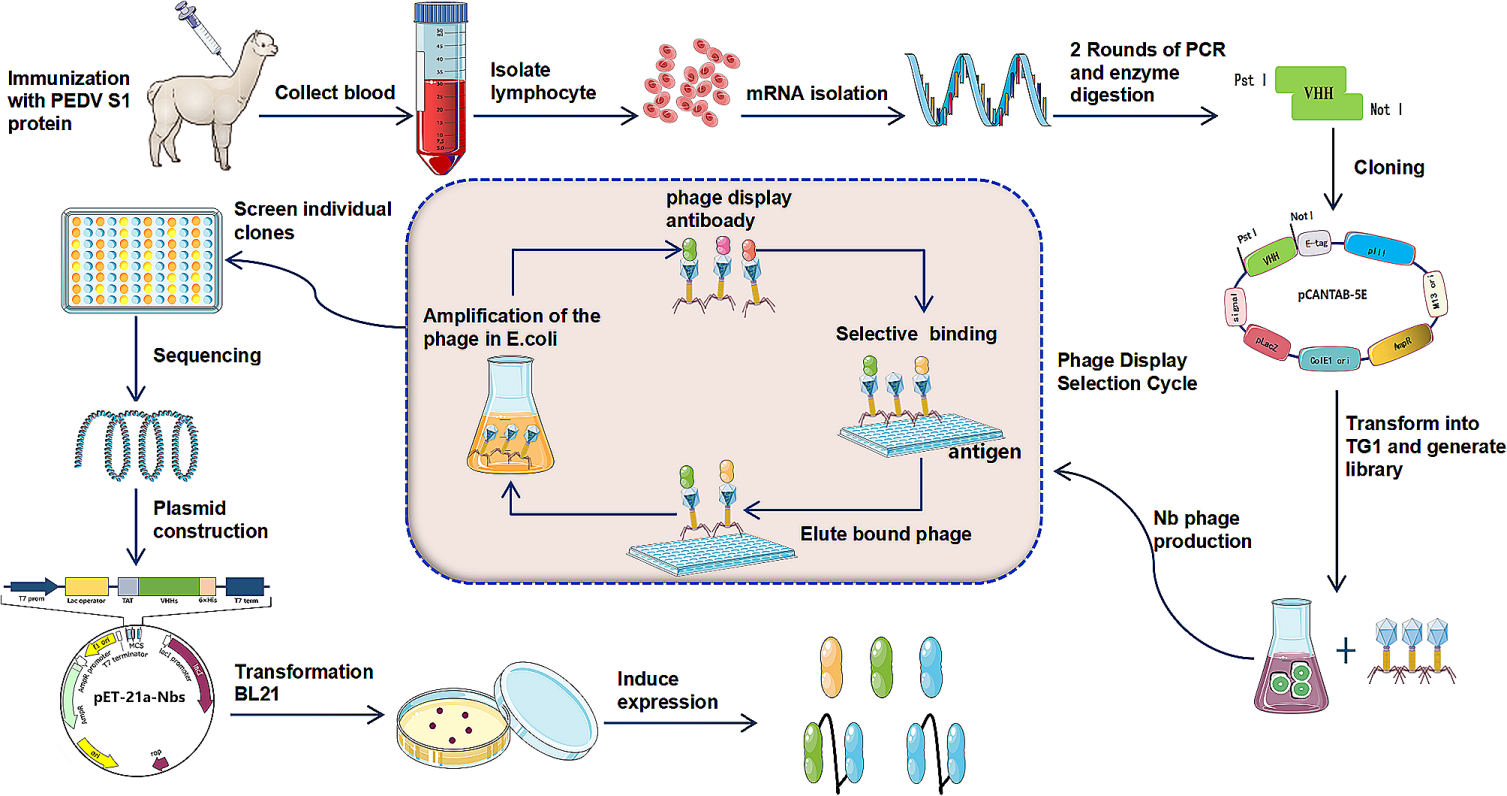
Schematic diagram of S1 protein specific nanobody screening

**Fig. 2:**
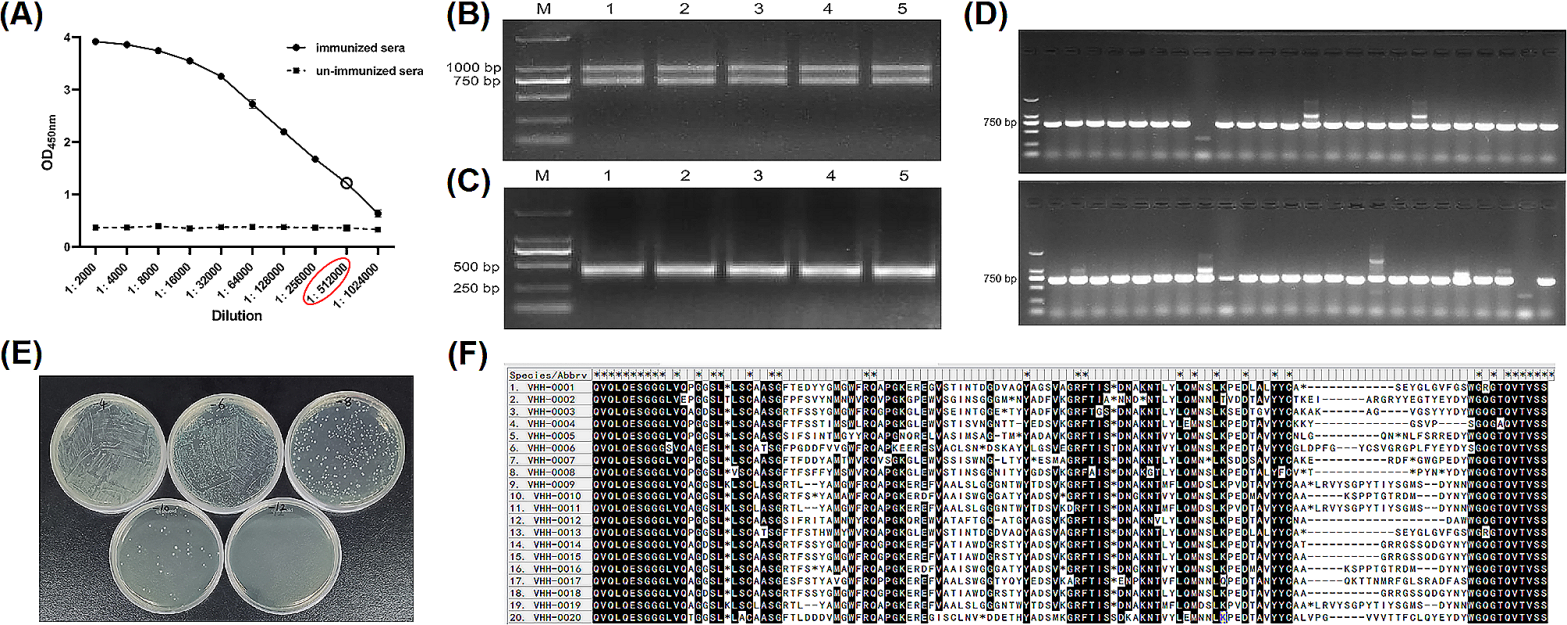
VHH library construction and quality assessment. (**A**) ELISA titer of anti-S1 antibody in the immune alpaca serum. (**B**) and (**C**) amplification of VHH gene by nested PCR. (**D**) Evaluate the positive rate of the VHH library by colony PCR. (**E**) VHH library capacity calculation. (**F**) Identification of sequence diversity in VHH libraries

The VHH library construction strategy is described in Fig. 1. After immunizing alpacas with the S1 protein five times, we collected the antiserum at a titer of 1:512,000, indicating a strong immunogenic response **(**Fig. 2-A**)**. We extracted total RNA from peripheral blood lymphocytes (PBLs) and reverse-transcribed it into cDNA. This cDNA was then used as a template for two rounds of Nested PCR to harvest a 400 bp fragment **(**Fig. 2-B and C). The VHH product was purified and ligated with the linearized pCANTAB-5E phagemid vector. The resulting mixture was electroporated into TG1 competent cells to generate a VHH library. The library was evaluated for quality, size, percentage inserts, and sequence diversity. The size of the library was approximately 6.4 × 10^13^**(**Fig. 2-E**)**, with a percentage insert of about 96% **(**Fig. 2-D**)**, and rich sequence diversity **(**Fig. 2-F**)**.

### Specific nbs panning and enrichment

The titer of the recombinant phage library reached 9.0 × 10^12^ pfu/mL after the rescue with the M13KO7 helper phage. Three consecutive rounds of bio-panning were performed to attain highly specific nanobodies against the PEDV S1 protein, using the protein itself or PBS as the antigen. The results, as displayed in Table 1, showed a gradual elevation in the recombinant phage recovery rate after three rounds of screening. The enrichment factors were 5.6-, 207-, and 1117-fold higher after the first, second, and third rounds of panning, respectively, as compared to the negative control. This indicates the significant enrichment of specific nanobodies during bio-panning.

**Table 1:** Enrichment of S1-specific Nbs^a^ in the VHH library from three rounds of biopanning

Round of panning	Input phage(pfu/well)	*P* output (pfu/well)	*N* output (pfu/well)	Recovery (*P*/input)	*P*/*N*
1_st_	5 × 10^11^	2.4 × 10^6^	4.3 × 10^5^	4.8 × 10^− 6^	5.6
2_nd_	5 × 10^11^	6.2 × 10^7^	3.0 × 10^5^	1.2 × 10^− 4^	207
3_rd_	5 × 10^11^	7.6 × 10^8^	6.8 × 10^5^	1.5 × 10^− 3^	1117

^a^*P* output, the phage titers from the positive wells (PEDV S1); N output, the phage titers from the negative wells (PBS)

### Nbs screening by indirect ELISA

**Fig. 3:**
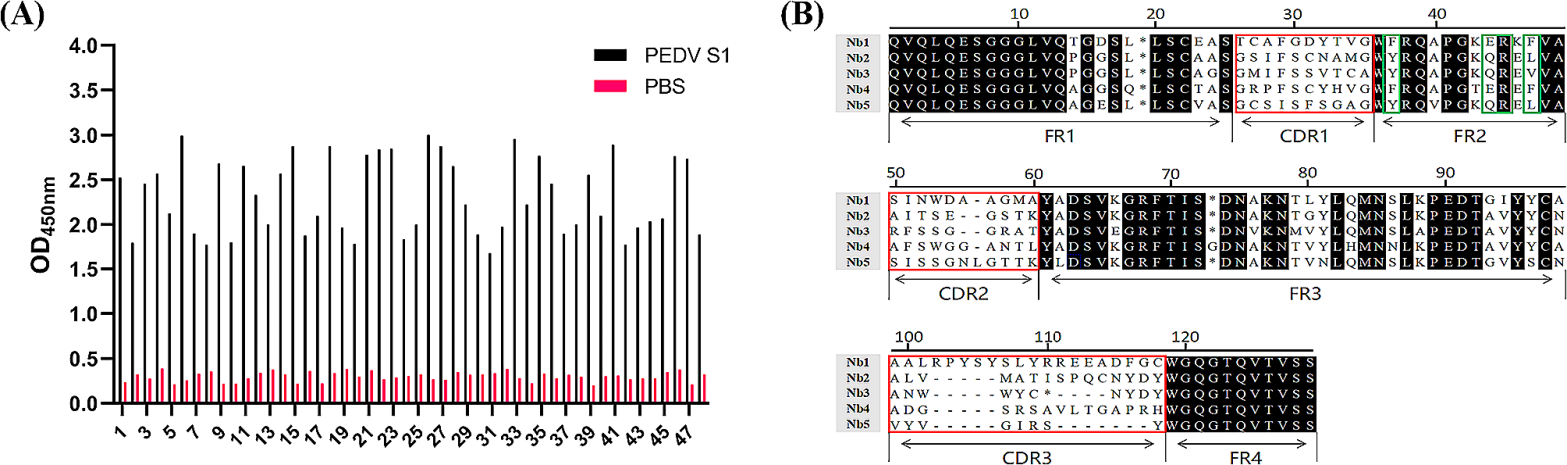
Screening Nbs against PEDV S1 protein. (**A**) Analysis of the binding ability of recombinant Nbs against S1 by inderect ELISA. (**B**) Functional region division and amino acid sequence analysis of 5 Nbs

To obtain specific and high-affinity clones against PEDV, phage ELISA was performed. We randomly selected forty-eight clones from the final round phage display library, and all selected clones were positive **(**Fig. 3-A**)**. The positive clones underwent sequencing analysis, where we classified them based on the amino acid sequence of the antibody hypervariable region. A total of five different Nbs were selected **(**Fig. 3-B**)**. The complementarity determining regions comprise hydrophilic amino acid substitutions at positions 37, 44, 45, and 47 in the relatively conservative FR2 framework. Additionally, all five Nbs contain cysteine residues within CDR1 and CDR3 regions, capable of forming disulfide bonds, thus contributing to the formation of a ring structure. This plays a crucial role in stabilizing the structure of the antigen-binding region of the nanobody. Three Nbs with high OD values were selected for further study and these Nbs were designated SF1, SF2 and SF3.

### Epitope analysis by additive ELISA

The OD_450nm_ values for the three anti-PEDV Nb were individually and jointly determined by additive ELISA, and the AI (Additivity index) values were calculated. The AI values of SF1 merged with SF2 and SF3 were 77.7% and 85.8%, respectively. SF2 paired with SF3 generated an AI value of 75.7%. Since the stacking indices of Nb are greater than 50%, confirming that each of the three Nbs recognizes different epitopes of PEDV **(**Table 2**)**. On this basis, SF2 and SF3 with the highest OD values were selected for tandem linkage and submitted to Sangon Biotech (Shanghai) Co., Ltd for biparatopic and bivalent nanobody recombinant vector construction, and sequentially named SF-B (SF2-SF3) and SF-E (SF3-SF3).

**Table 2:** Additivity index (^a^AI) measure by the additive ELISA

Nbs	SF1	SF2	SF3
	AI%	AI%	AI%
**SF1**	-	77.7	85.8
**SF2**	77.7	-	75.7
**SF3**	85.8	75.7	-

^a^AI was calculated using the equation: AI = [2A_1 + 2_/ (A_1_ + A_2_) − 1] × 100%, where A_1_ and A_2_ represent the OD_450nm_ values for each of two Nbs tested, and A_1 + 2_ represents the OD_450nm_ value when the two Nbs are mixed

### Expression and purification of nanobodies

**Fig. 4:**
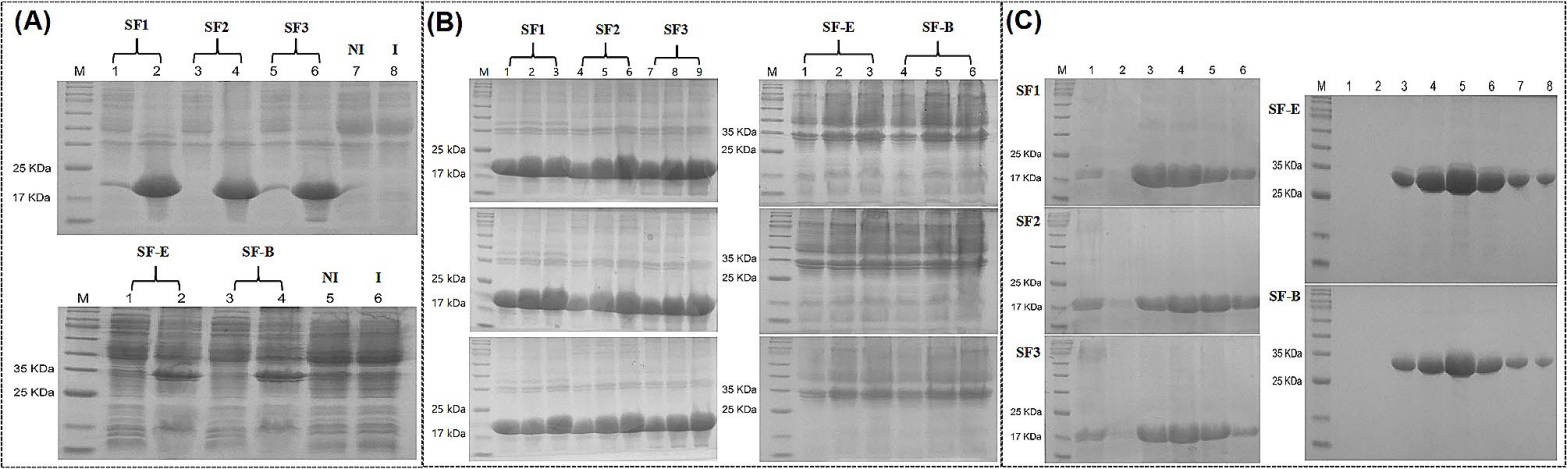
Expression and purification of Nbs recombinant protein in *E. coli.* (**A**) Analyze the expression of Nbs recombinant protein as inclusion bodies by SDS-PAGE, upper panel, lane M: Solarbio 180 Marker; lanes 1,3 and 5: supernatants of bacterial lysates; lanes 2, 4 and 6: precipitates of bacterial lysates; NI: non-induced empty vector bacteria; I: induced empty vector bacteria. lower panel, lanes 1, and 3: supernatants of bacterial lysates; lanes 2 and 4: precipitates of bacterial lysates; other lanes are the same as upper panel. (**B**) Optimization of conditions for induction of recombinant Nbs protein. upper panel, optimization of IPTG concentrations; middle panel, optimization of IPTG inducing time; lower panel, optimization of IPTG inducing temperature. (**C**) Purification of Nbs recombinant protein

The monovalent nanobody proteins SF1, SF2, and SF3, with an approximate 19 kDa, and the multivalent nanobody proteins SF-E and SF-B, with an approximate 33 kDa, were all expressed in inclusion bodies detected by 12% SDS-PAGE **(**Fig. 4-A**)**. The protein induction and expression conditions were optimized and ultimately, a concentration of 0.5 mM of IPTG (isopropyl-b-d-thiogalactopyranoside), an induction time of 12 h, and an induction temperature of 37℃ were selected as the final conditions **(**Fig. 4-B**)**. After conducting extensive induction and sonication, high-purity Nbs proteins were acquired through purification using a Ni-NTA affinity chromatography column. The purity of these proteins was assessed by 12% SDS-PAGE **(**Fig. 4-C**)**. Following denaturation, the proteins were dialyzed in dialysate with decreasing concentrations of urea. The proteins ultimately reached concentrations of 1.13 mg/mL, 1.25 mg/mL, 1.36 mg/mL, 2.2 mg/mL, and 1.8 mg/mL, as determined by the BCA assay.

**Fig. 5:**
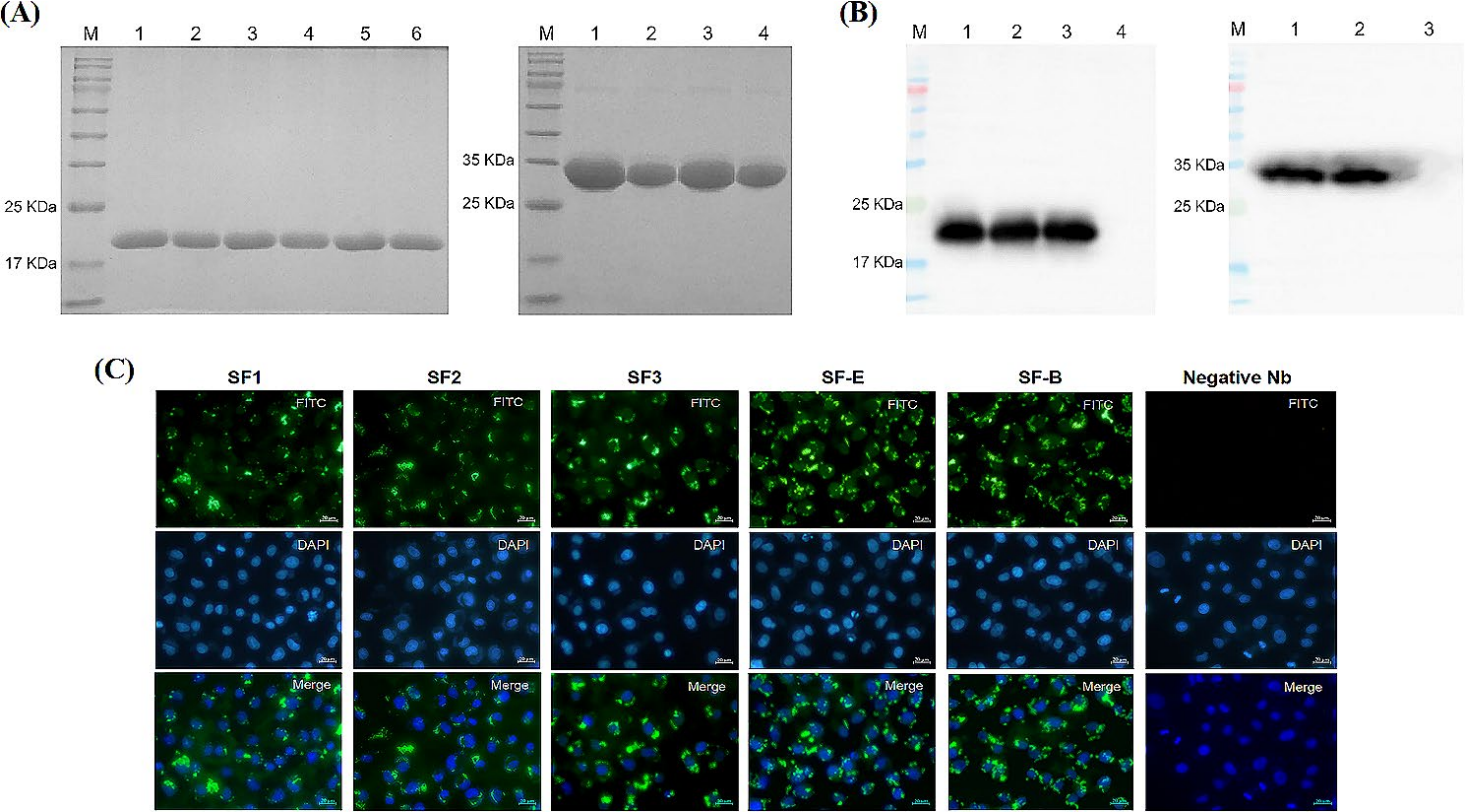
Expression and identification of the recombinant nanobodies. (**A**) Results of 12% SDS-PAGE. left panel, lane M: Solarbio 180 Marker; lane 1, 3 and 5 represent SF1, SF2 and SF3 before renaturing; lane 2, 4 and 6 represent SF1, SF2 and SF3 after renaturing. right panel, lane M: Solarbio 180 Marker; lane 1 and 3 represent SF-E and SF-B before renaturing; lane 2 and 4 represent SF-E and SF-B after renaturing. (**B**) Results of WB. Left panel, lane M: Solarbio 180 Marker; lane 1 to 3 represent SF1, SF2 and SF3; lane 4: bacteria not induced by IPTG. Right panel, lane M: Solarbio 180 Marker; lane 1 and 2 represent SF-E, SF-B; lane 4: bacteria not induced by IPTG. (**C**) Nanobodies bound to PEDV-infected cells. PEDV-infected Vero cells were incubated with nanobodies at 24 h post-infection and then stained with the FITC conjugated anti-His monoclonal antibody. Scale bar = 20 μm

As validated by 12% SDS-PAGE and western blotting. The results showed that the proteins displayed high purity, with minimal loss during the renaturation process, and all of them reacted with rabbit anti-His tag antibody **(**Fig. 5-A and B). To confirm whether purified Nbs specifically bound to PEDV, an IFA was conducted. The result showed that SF1, SF2, SF3, SF-E and SF-B stained PEDV-infected Vero cells but did not react with negative Nb, indicating that purified Nbs specifically bound to PEDV **(**Fig. 5-C**)**.

### VHH biological characterization

**Fig. 6:**
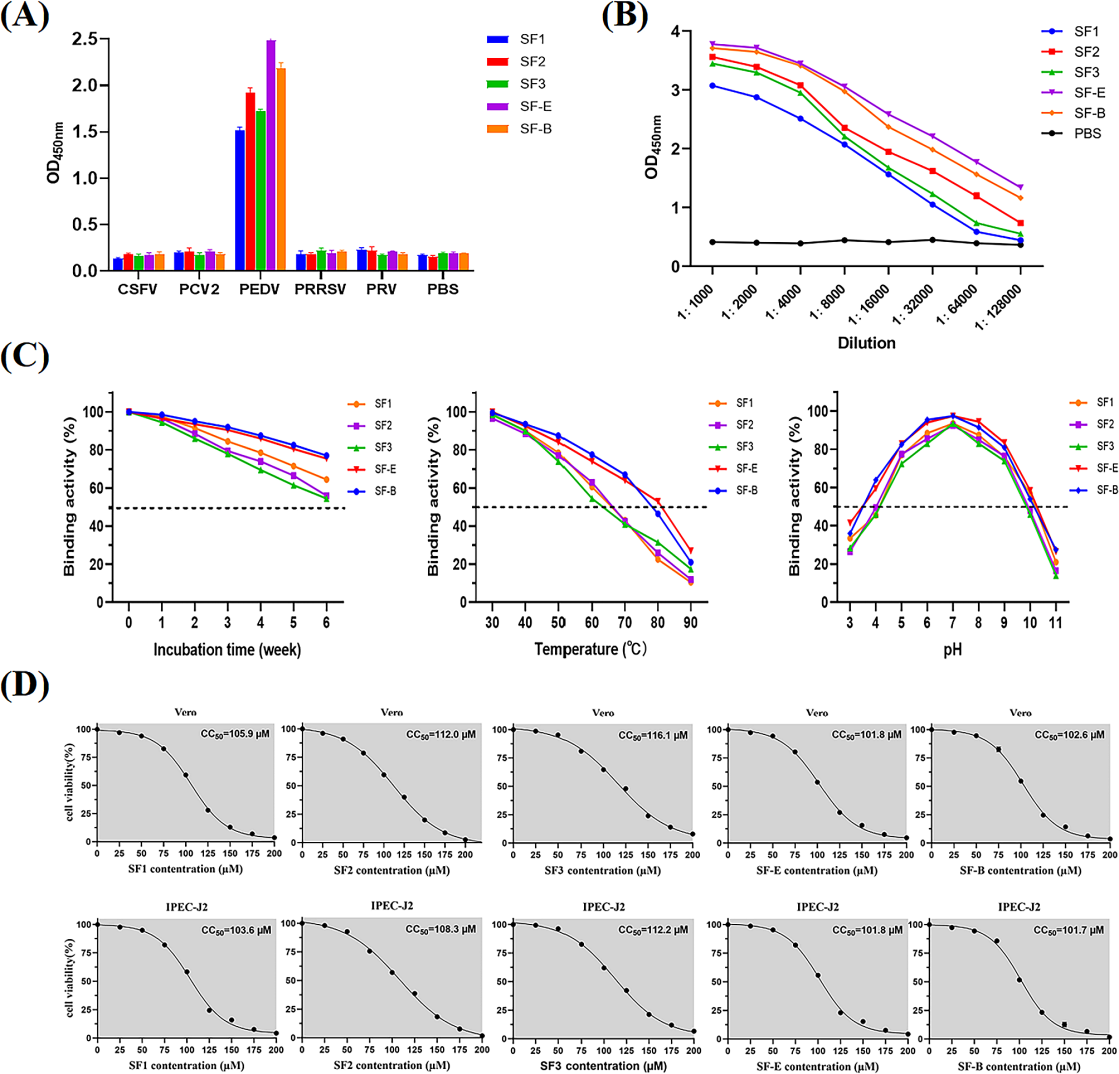
Comparative analysis of the biological properties and cytotoxicity. (**A**) Specificity analysis of nanobodies against PEDV. (**B**) Identification of nanobody stability affinity. (**C**) Stability of anti-PEDV nanobodies analyzed by indirect ELISA. (**D**) Cytotoxicity of anti-PEDV nanobodies analyzed by CCK-8

The reaction of the Nbs with PEDV, CSFV, PCV2, PRRSV and PRV was investigated by indirect ELISA. The Nbs exhibited high specificity in binding to PEDV **(**Fig. 6-A**)**. The analysis of the Nbs showed that SF1 and SF3 had a binding potency of 1:32,000, SF2 had a binding potency of 1:64,000, and SF-E and SF-B had a binding potency of 1:128,000, all of which showed high affinity **(**Fig. 6-B**)**. The stability analysis of the Nbs demonstrated that after six weeks of storage at 4 ℃, all the Nbs maintained over 50% of their binding activity. Additionally, all five Nbs exhibited high temperature and pH tolerance, with the multivalent nanobodies demonstrating significantly greater stability than the monovalent nanobodies **(**Fig. 6-C**)**.

### Evaluation of the in vitro cytotoxicity of Nbs

The nanobody’s cytotoxicity was measured using the CCK-8 assay. 2-(2-methoxy-4-nitrophenyl)-3-(4-nitrophenyl)-5-(2,4-disulfophenyl)-2 H-tetrazole sodium salt (WST-8) can be reduced by mitochondrial dehydrogenase in the presence of an electronically coupled agent to an orange-yellow dye rich in methanol. The number of viable cells was quantified using dynamic colorimetric methods. Nanobodies were added to Vero and IPEC-J2 cells and incubated for 24 h. The WST-8 amount was measured to determine the reduction of the methyl dye. The results indicated that all five nanobodies had CC_50_ values exceeding 100 µM, which demonstrates their safety **(**Fig. 6-D**)**.

### Neutralization effect of Nbs to PEDV

**Fig. 7:**
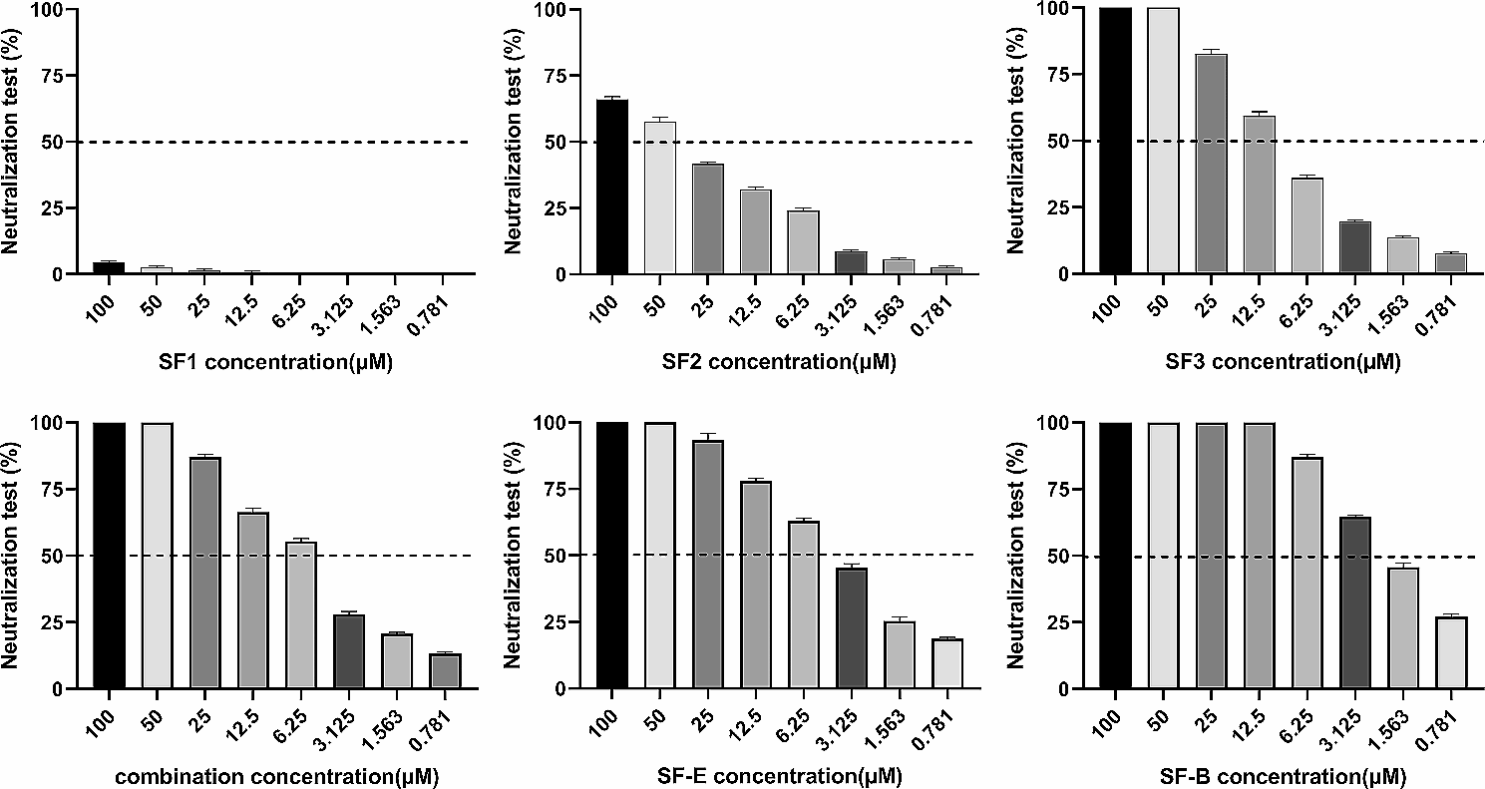
Naturalization activities of anti-PEDV strain SD2020. The combination is a mixture of SF1, SF2, and SF3 nanobodies of uniform final concentration

To determine whether these five nanobodies neutralized PEDV infection, we performed microplate neutralization experiments. Two serial dilutions of the Nbs working stocks (100-0.781 µM) were tested in triplicate. The findings demonstrate that SF1 did not neutralize PEDV. However, SF3 and SF-E were effective in neutralizing the virus at 50 µM, and SF-B was effective in neutralizing the virus at a lower concentration of 12.5 µM **(**Fig. 7**)**. We conclude that SF3, SF-E and SF-B are potential candidates for PEDV therapy. The range of IC_50_ values for monovalent nanobodies was 6.25-50 µM, while that for multivalent nanobodies was 1.563–6.25 µM. Multivalent nanobodies outperform monovalent nanobodies significantly in neutralization and possess the potential to neutralize viral infection in vitro.

### Nbs inhibit PEDV replication in vero cells

**Fig. 8:**
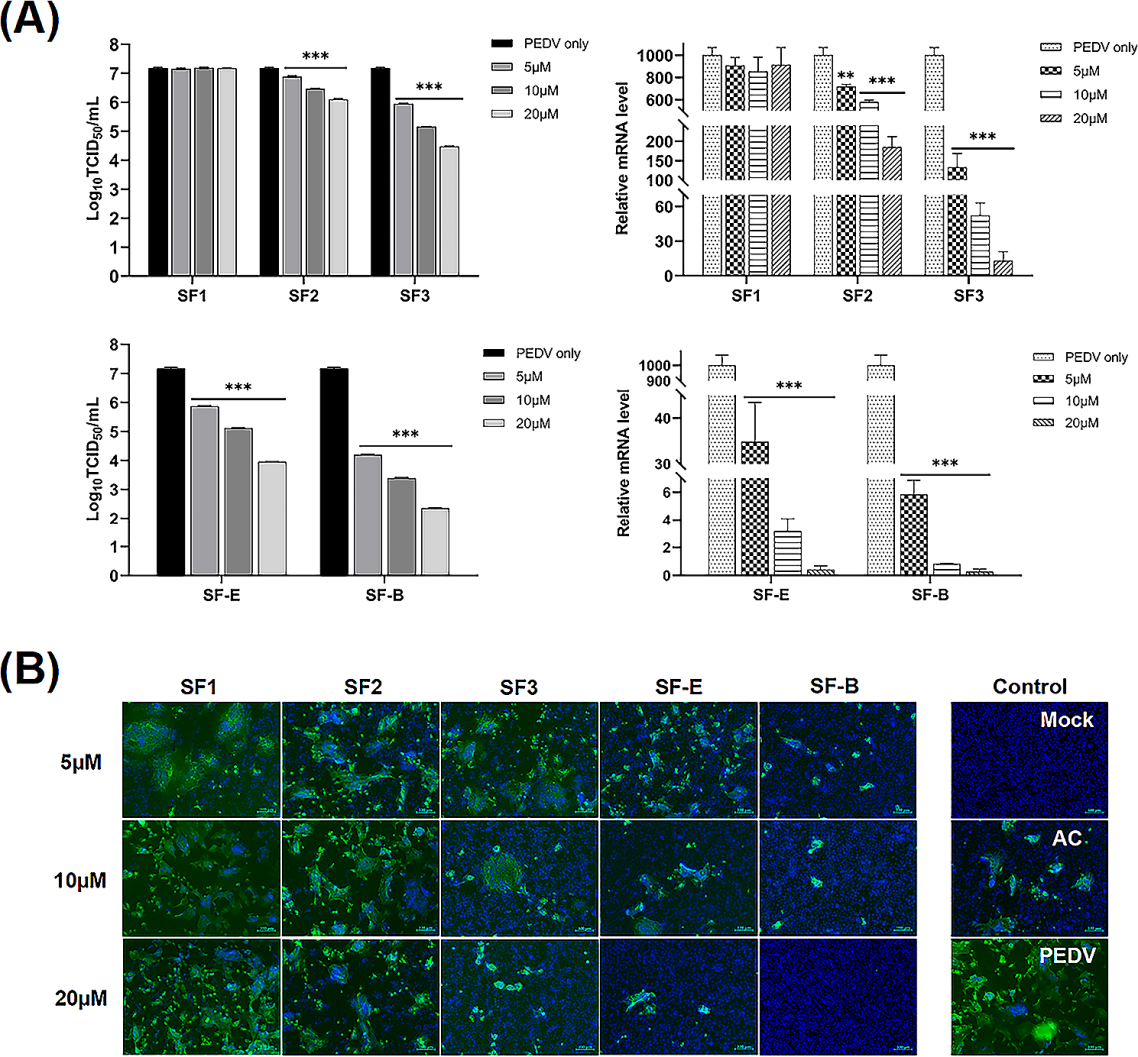
Nanobodies inhibits PEDV replication in Vero cells. (**A**) Right panel, Vero cells were seeded in plates and incubated with 0, 5, 10, or 20 µM nanobodies for 2 h and then infected with PEDV (1000 TCID_50_). At 36 h, the expression of PEDV N protein mRNA was determined by qRT-PCR; Left panel, supernatant viral titers were analyzed using TCID_50_. (**B**) Vero cells were treated as described for panel A. Infection of cells was determined by IFA. GAPDH was used as an internal control to normalize the quantitative data. Scale bar = 100 μm. Data are expressed as the means ± standard deviations (SD) of the results of three independent experiments. *p* values were calculated using Student’s *t* test or analysis of variance (ANOV A). *, *p* < 0.05; **, *p* < 0.01; ***, *p* < 0.001; ns, not significant

Vero cells were selected to evaluate the antiviral effects of five nanobody strains. Vero cells were treated with 0, 5, 10, or 20 µM Nbs and then infected with 1000 TCID_50_/mL PEDV. Cells and culture supernatants were harvested after 36 h to evaluate the effect of Nbs on PEDV replication. The results showed that the relative mRNA levels of PEDV N protein and progeny virus in culture supernatants were significantly decreased after treatment with Nbs (SF2, SF3, SF-E, SF-B) compared to the medium control **(**Fig. 8-A**)**. The effect became increasingly evident as the concentration of nanobody treatment was increased in a concentration-dependent manner. However, SF1 showed no inhibitory effect on N protein mRNA and supernatant progeny virus titers **(**Fig. 8-A**)**. The IFA results also revealed that Nbs, but not SF1, suppressed PEDV S1 protein expression in a dose-dependent manner **(**Fig. 8-B**)**. These results suggest that Nbs effectively interrupted PEDV replication. Among them, excellent antiviral properties were demonstrated by the multivalent nanobodies SF-E and SF-B.

**Fig. 9:**
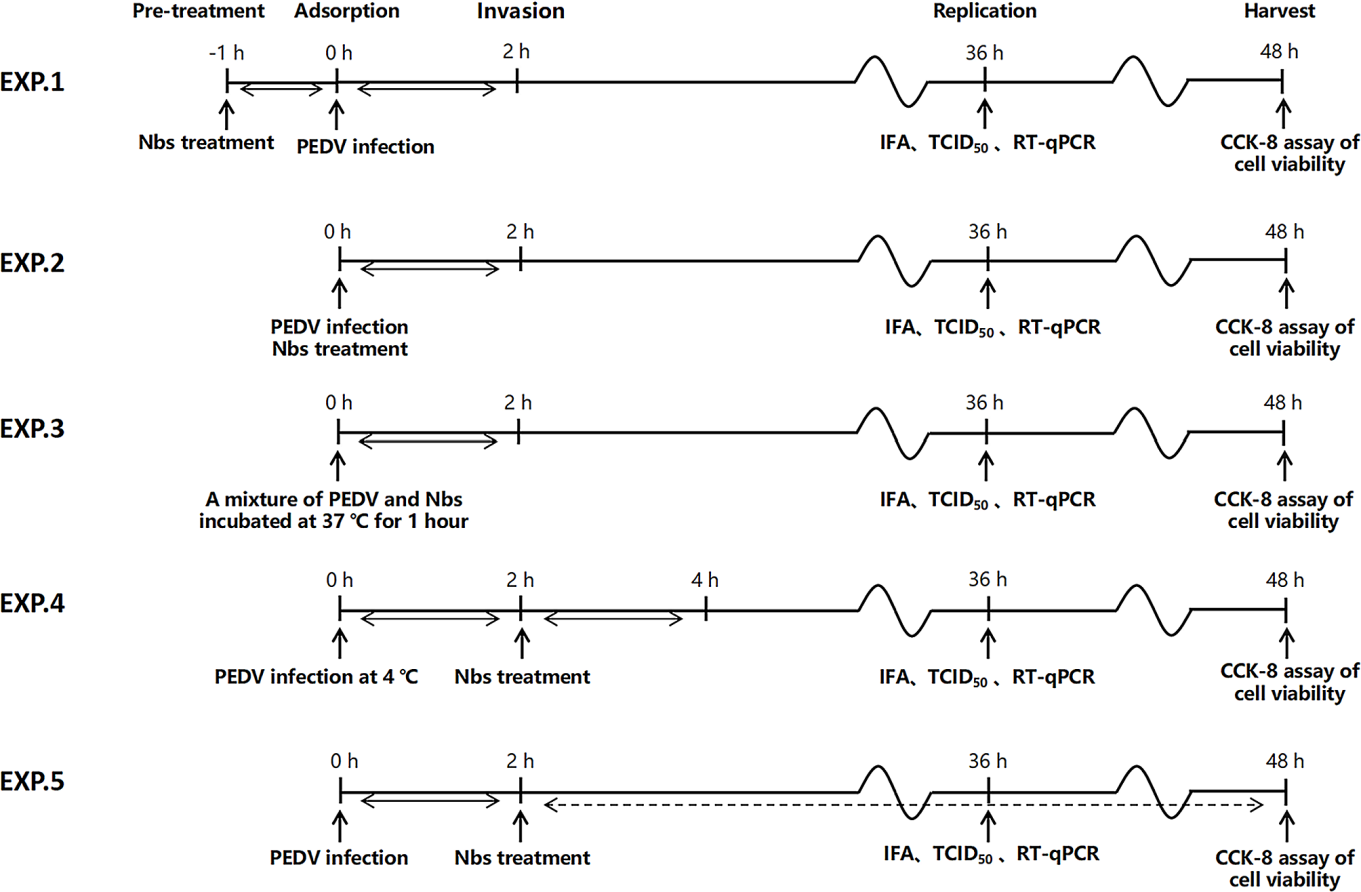
Schematic of application of nanobodies at different time of PEDV infection

**Fig. 10:**
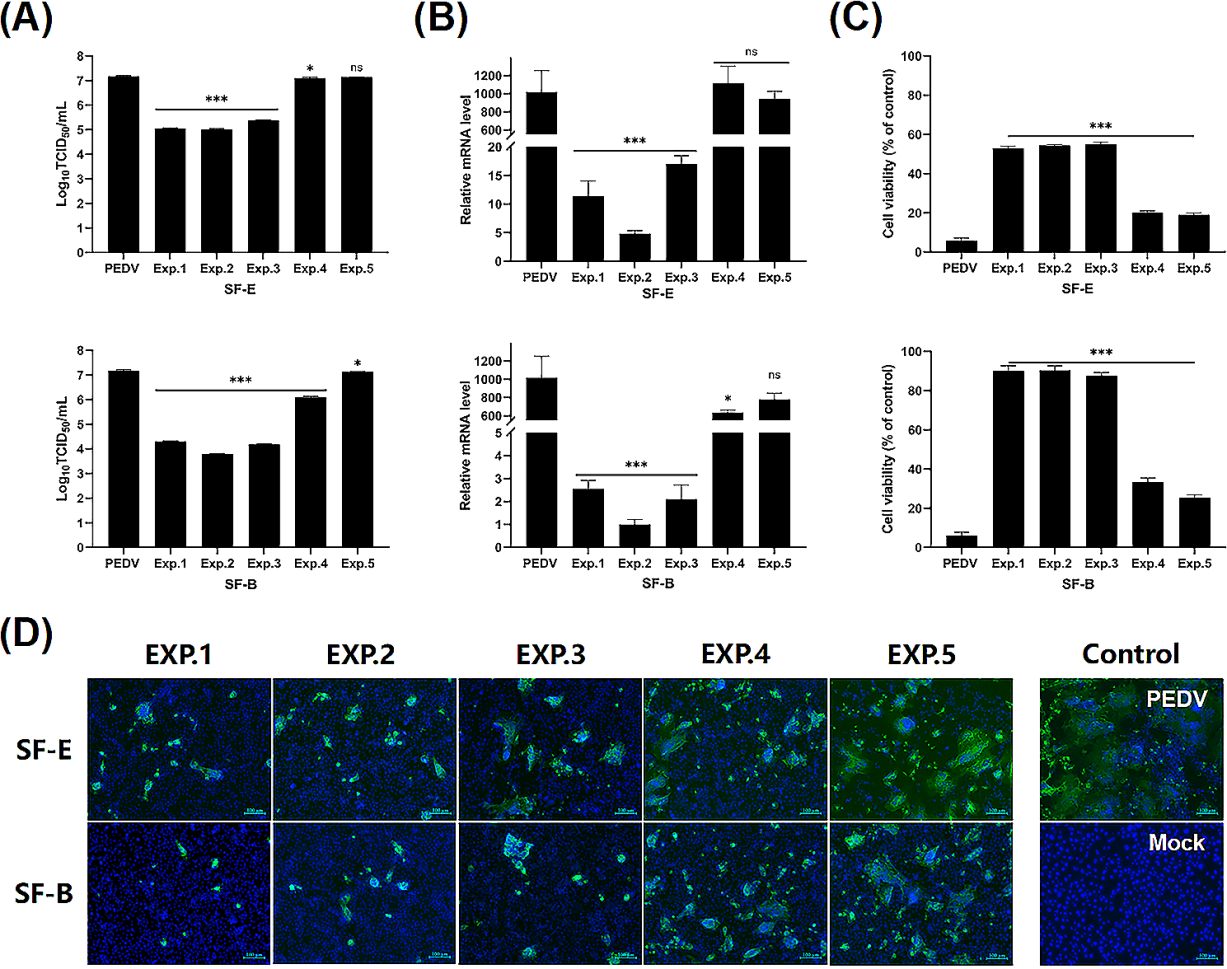
Mechanisms of multivalent nanobodies on PEDV proliferation. Vero cells were seeded in plates and incubated with five treatments performed before and after virus infection. At 36 h, the expression of PEDV N protein mRNA was determined by qRT-PCR (**B**); supernatant progeny viral titers were determined by TCID_50_ (**A**); infection of cells was determined by IFA (**D**), Scale bar = 100 μm. At 48 h, cell survival after 48 h of PEDV infection (**C**). GAPDH was used as an internal control to normalize the quantitative data. Scale bar = 100 μm. Data are expressed as the means ± standard deviations (SD) of the results of three independent experiments. *p* values were calculated using Student’s *t* test or analysis of variance (ANOV A). *, *p* < 0.05; **, *p* < 0.01; ***, *p* < 0.001; ns, not significant

At 4 °C, viruses can only bind to cells and do not invade them. However, when the ambient temperature increases to 37 °C, viruses become capable of invading cells. Therefore, we investigated whether multivalent nanobodies function effectively against the PEDV virus when it adsorbs to cells or invades into cells. Additionally, we investigated the preventive and therapeutic potential of nanobodies against the PEDV virus following treatment of cells with five different approaches, as illustrated in Fig. 9. The TCID_50_ results revealed that SF-E and SF-B were able to significantly reduce the titer of progeny virus when they were applied to the virus adsorption and invasion. Both constructs of nanobodies were capable of significantly reducing the titer of progeny virus after pretreatment of cells that also had virus infection. However, when nanobody treatment was administered following virus invasion of the cells, SF-B was found to significantly reduce the progeny virus titer, whereas SF-E had no significant impact **(**Fig. 10-A**)**. The qRT-PCR results indicate that treatment with SF-B during viral adsorption and cell invasion resulted in a significant reduction of the relative RNA expression level of the virus, whereas treatment with SF-E during viral invasion did not significantly reduce the titer of the virus progeny. Treating cells with nanobodies significantly inhibited relative RNA expression of the virus. However, both nanobody preparations did not significantly affect viral gene expression when cells were treated after viral infection **(**Fig. 10-B**)**. The IFA obtained outcomes that are comparable to the experimental findings mentioned earlier **(**Fig. 10-D**)**. These results indicate that nanobodies can play an inhibitory role in both viral adsorption and cell invasion. The S protein plays a crucial role in attaching, entering, binding to receptors, and fusion with the cell membrane, while the Nb-S dimer complex may disrupt the correct protein conformation of the S protein necessary for viral invasion of the cell, and thus abrogating viral replication. All five treatments significantly increased cell viability 48 h after viral infection **(**Fig. 10-C**)**. SF-B will be further utilized for subsequent animal experiments.

## Discussion

Humoral responses are crucial for protecting neonatal pigs against PEDV infection [23]. The traditional method of safeguarding piglets from PEDV infection is through immunizing pregnant sows with inactivated or attenuated viruses [24]. The immune response enters the mammary gland through the gut-mammary gland-sIgA axis, which results in passive lactogenic immunity in suckling piglets [25]. While this procedure offers efficient safeguarding for newborn piglets, research has revealed that not all vaccinated sows develop lactogenic immunity for protection, while piglets remain vulnerable to PEDV post-weaning [3]. Because of the genome’s high variability, no commercial vaccines can provide complete immunity [26]. Additionally, effective drugs for the treatment of PED are not available. Passive immunization with exogenous antibodies may emerge as a promising strategy for preventing diarrheal diseases in piglets [27]. Administration of chicken-derived anti-PEDV egg yolk immunoglobulin (IgY) to piglets led to a significant decrease in mortality rate following an attack [16]. Furthermore, several monoclonal antibodies against PEDV have been reported in studies, all of which neutralize viral infection [28, 29]. Although high-affinity monoclonal antibodies have received priority attention as potential therapeutic agents in research and clinical applications, the expense has limited their use [30].

Considering the unique properties of Nbs, they have been widely used in the development of therapeutic antibody drugs, molecular imaging, diagnostic reagents, targeted release of drugs, and various other areas of scientific research [31]. Compared to monoclonal antibodies, Nbs are structurally straightforward, functional even without modifications, can function without the Fc domain, and feasible for large-scale expression in bacteria and yeast [32]. During the production of antibodies, an antigen with higher immunogenicity possesses a greater potential for inducing an immune response. Recombinant proteins expressed in eukaryotic cells can undergo post-translational modifications and molecular folding, improving the ability of antibodies to recognize neutralizing epitopes [33]. In this study, we obtained S1 proteins close to the natural conformation using the CHO expression system, which helped induce an immune response in the alpacas so that a high-quality library could be made. It also helped with the selection of the binders. After three rounds of biopanning, five Nbs with high affinity to PEDV were successfully selected. We then performed a comparative analysis of the amino acid sequences of the Nbs, which revealed differences primarily concentrated in the complementarity determining region. Apparent amino acid deletions and insertions were observed in the CDR3 region of the Nbs. The CDR3 region of VHH has a large exposed area and its CDR1 region provides flexibility that allows the Nbs to bind to both antigen surfaces and antigenic gap epitopes not recognized by standard antibodies [34]. The differences in CDR diversity of the VH structural domains may reflect the fact that these Nbs may bind different epitopes, and the results of additive ELISA experiments validated our hypothesis.

Cloning two Nb genes in a tandem will generate bivalent, biparatopic, or bispecific constructs, depending on whether: (1) the genes encoding two identical Nbs, (2) the genes encoding two different Nbs targeting different epitopes on the same antigen, or (3) the genes encoding two Nbs against different antigens are cloned in tandem, respectively [35]. By targeting dual targets, blocking signaling pathways and exerting unique or overlapping functions, effectively preventing immune escape, and possessing the advantages of higher specificity, better targeting and reduced off-target toxicity, they greatly expand the boundaries of biological therapy and have been the hotspot of antibody engineering research in recent years [36, 37]. Dong et al. showed that the combination of specific nanobodies had a synergistic effect in blocking the interaction of Spike proteins with ACE2 receptors and enhanced the neutralization of SARS-CoV-2 infection [38]. In the next step, He et al. [39]. built dimeric and trimeric nanobodies that showed higher ability than monomeric nanobodies in different MERS-COV strains. The multimer potently blocked the RBD domain of MERS-CoV from attaching to the cell surface receptor (DPP4) and prevented entering of the virus in the animal’s cell. In another attempt, Schoof et al. [40]. isolated several nanobodies with high affinity for different epitopes of the SARS-CoV-2 spike protein, from which multivalent nanobodies with significantly improved inhibitory activity against the SARS-CoV-2 virus were derived. Based on the above theoretical research and practice, in this study, we tandemized the nanobodies with Gly4Ser flexible linkers (repeat three times) to obtain biparatopic and bivalent nanobodies, and compared and evaluated their antiviral infection effects.

The current results indicate that multivalent nanobodies may offer a more effective means of preventing viral replication. This is likely due to their higher affinity and the increased spatial resistance resulting from multivalent couplings. In future studies, we intend to prepare more tandems or couples with Fc portion of IgG and evaluate their blocking effect on PEDV replication. We constructed a total of five Nbs and verified that the Nbs inhibited the replication of PEDV strains in Vero cells, providing a theoretical basis for the use of Nbs as antiviral drugs. Although SF1 bound specifically to PEDV, it had no antiviral activity. Additive ELISA tests confirmed that all three Nbs bind to different epitopes of the S1 protein. The different recognition epitopes are thought to be the main reason for the difference in neutralizing efficacy of the Nbs. The S1 protein is responsible for cell attachment and the S2 protein mediates fusion of the virus with the host membrane. Binding of nanobodies to the S1 protein alters the original conformation, thereby affecting the infection process of the virus. The experimental results showed that the nanobodies mainly inhibited viral adsorption, and surprisingly, SF-B seemed to play a role in viral invasion of the membrane fusion junction. Unfortunately, in the present study we did not identify the interaction sites between SF-B and the PEDV S1 protein, which may be helpful in clarifying the molecular mechanism by which Nb blocks PEDV replication. This aspect requires further in-depth study. The use of Nbs as molecular chaperones for protein crystallization and the resolution of the crystal structure of the S1 protein may provide an important reference for the design of antiviral drugs. This will be included in our next research direction to focus on.

In the next step, we will test the protective effect of SF-B in neonatal piglets. Oral administration could serve as a promising therapeutic strategy, several studies have developed Nbs for oral delivery in animal feed to control pathogenic bacteria in food production animals [41]. Nonetheless, the significant protease sensitivity of nanobodies in the gastrointestinal (GI) tract presents a crucial limitation for oral administration. The biophysical characteristics of nanobodies can be modified to enhance their resilience to proteases, for instance, by introducing cysteine residues to generate additional disulfide bonds, and mutations at protease-sensitive sites [42]. Alongside genetic alteration of the nanobodies, selecting a suitable targeted delivery system is also essential. Bacteria utilized in food products have also been utilized recently for their capacity to deliver recombinant proteins in situ (such as secreted or wall-anchored) to maintain product activity after gastric transit [43]. One potential benefit of in planta Nb expression, as opposed to microbial production of soluble proteins, is that Nbs delivered in a plant matrix are protected against proteolytic degradation in the GI tract [44]. Furthermore, plants can assemble complex Nb constructs in vivo, and this is an advantage compared to prokaryotic hosts which cannot easily assemble proteins consisting of multiple polypeptides. The findings indicate increased stability of Nbs expressed in planta because heat-treated (90 °C) Nbs were able to neutralize the virus [45]. For such plant-based delivery systems to be efficient, it is crucial to ensure adequate quantitative release of the product from the plant matrix in the GI tract, which should be a significant research priority for the future [46]. Furthermore, elaboration is necessary concerning the inclination of Nbs in inducing escape mutants and their mechanism of action on the microbiota of the gut.

## Conclusion

In this study, a set of potent neutralizing and immunized PEDV Nbs were identified from an immunized alpaca library. These Nbs neutralizing and immunized PEDV infection by binding to different epitopes of the S1 protein. Subsequently, biparatopic nanobodies were engineered through covalent linkage of the Nbs, which further improved neutralizing capacity. In summary, nanobodies can be used as a potential therapeutic and prophylactic drug for PEDV.

## Materials and methods

### Cells, viruses and animals

The Vero cells were propagated in Dulbecco’s modified Eagle’s medium (DMEM; Hyclone) supplemented with 10% fetal bovine serum (FBS; Gibco). Cells were cultured at 37℃ in a 5% CO_2_ incubator and passaged approximately every 48 h. Porcine epidemic diarrhea virus (PEDV) strain SD2020(GenBank ID: OP894120.1)was isolated by Shandong Key Lab of Preventive Veterinary Medicine, Qingdao Agricultural University (QAU), China. PEDV was proliferated on Vero cells and stored at -80℃ until further use. To immunize alpaca, the virus was inactivated with a final concentration of 0.1% β-propiolactone at 4 °C for 24 h, followed by incubation in a water bath at 37 °C for 2 h to prepare the inactivated virus for immunized alpaca. Porcine reproductive and respiratory syndrome virus (PRRSV), classical swine fever virus (CSFV), pseudorabies virus (PRV), porcine circovirus type 2 (PCV2), PEDV S1 protein was expressed based on a CHO expression system and maintained at the Shandong Key Lab of Preventive Veterinary Medicine. Alpacas are bred by Qingdao Agricultural University Teaching Experimental Base.

### VHH sequence acquisition

A healthy adult male alpaca was immunized with 2 mL of PEDV S1 recombinant protein and 4 mL of inactivated virus emulsified at a ratio of 1:1 (v/v) mixture with ISA 201 adjuvant (SEPPIC, France; 7014234-6). A total of five immunizations were given at two-week intervals. Serum was collected one week after each immunization for antibody potency testing. Peroxidase-conjugated AffiniPure goat anti-Alpaca IgG H&L (Stratech, UK; 128-035-003-JIR) was used as the secondary antibody for titer measurement of ELISA. After immunization was complete and the alpacas were returned to the base for further breeding, fresh blood was collected from which peripheral blood lymphocytes (PBLs) were isolated. Total RNA was isolated from PBLs using RNeasy^®^ Mini Kit (QIAGEN, Germany, Cat. No. 74,104) according to the manufacturer’s instructions, and the RNA was then reverse-transcribed to cDNA using the HiScript^®^ III 1st Strand cDNA Synthesis Kit (Vazyme, China; R312-01). The VHH genes were amplified by nested PCR using KOD One™ PCR Master Mix (TOYOBO, Japan). The PCR products were separated by electrophoresis and extracted from the gel using Gel Extraction Kit (QIAGEN, Germany, Cat. No. 20,021).

### VHH library construction

The pCANTAB-5E phagemid vector and purified VHH genes underwent digestion with *PstI* and *NotI* restriction enzymes (New England Biolabs, NEB) and were subsequently ligated with T4 DNA ligase (New England Biolabs, NEB) at 16 °C. One milliliter of *E. coli* competent TG1 cells should be taken, followed by the addition of 5 µg of recombinant plasmid. The mixture should then be gently mixed, and 100 µL should be added to electrocution cups that have been pre-cooled on ice. Each electrocution cup should contain 100 µL per cup. The electroporator’s parameters should be set to 2.5 kV, 5 ms. Immediately following the application of the shock, the shock cups should be removed, 1 mL of pre-warmed 2YT medium at 37 °C should be added, the cells should be resuspended, and they should be transferred to a sterile 50 mL centrifuge tube. This process should be repeated in order to complete the remaining shock transformation. The transformation products were incubated on a shaker at 37 °C and 200 rpm with shaking for one hour. The cells were incubated overnight at 37 °C on LB/AMP-GLU agar plates (100 µg/mL ampicillin and 4% glucose). The colonies were scraped and stored in LB with 20% glycerol at -80 °C [47]. The evaluation of library size and diversity was conducted by calculating the number of colonies and insertion rate through PCR amplification after gradient dilution. Positive clones were subsequently sequenced.

### Phage preparation and titration

Recombinant TG1 cells containing the pCANTAB-5E-VHH plasmid were inoculated into 2×YT/AMP-GLU medium with vigorous shaking at 200 rpm and 37 °C until OD_600nm_ of the bacterial culture reached approximately 0.6. After that, the M13KO7 helper phage(MOI = 20) was added to TG1 cells and incubated statically at 37 °C for 30 min. Cells were harvested by centrifugation at 2800*×g* for 10 min, resuspended, and grown overnight in 100 mL 2×YT/AMP-KAN medium (100 µg/mL ampicillin and 50 µg/mL kanamycin). Cell culture was collected by centrifugation at 4000*×g* for 30 min at 4 °C, and the supernatant was mixed with 20 mL PEG/NaCl. Phages were precipitated by centrifugation at 6000*×g* for 30 min after 5 h incubation on ice. Finally, the phage particles were resuspended in 1 mL of phosphate-buffered saline (PBS) and incubated overnight at 4 °C to completely solubilize the phage particles. TG1 cells were infected by taking 20 µl of phage solution at 10-fold specific dilution and incubated overnight on LB/AMP-GLU agar plates. The next day, the phage was quantified and the remaining phage was stored at -20 °C for subsequent experiments.

### Solid-phase panning

A 96-well plate was coated with PEDV S1 protein (100 µg/mL) overnight at 4 °C, and PBS was used as a control. After blocking with 1% BSA (200 µL/well) at 25 °C for 1 h, resuspended phage particles diluted in 1% BSA were prepared at a concentration of 5.0 × 10^12^ pfu/mL and incubated in panning plates (100 µL/well) at 25 °C for 1 h. After ten washes with PBS, elution was performed with 0.1 M triethylamine (100 µL/well) for 10 min at 25 °C, followed by neutralization with 1 M Tris-HCl (pH 7.4). A 4 mL aliquot of fresh exponentially growing *E. coli* TG1 culture was infected with a 400 µL aliquot of eluted phage particles for 30 min at 37 °C without shaking, followed by incubation in 100 mL 2×YT/AMP-GLU medium until the OD_600nm_ of the culture reached approximately 0.6. The M13KO7 helper phage was added to the TG1 cells to rescue and enrich the specific phage particles by three consecutive rounds of bio-panning with the above procedures. Enrichment in each round of panning was assessed by phage titration.

### Detection of specific nanobody

A total of 48 different colonies were randomly selected from the third round of panning and cultured in 96-well plates containing 100 µL of LB/AMP-GLU agar medium for 4 h at 37 °C. Subsequently, 20 µL of each cloned culture was transferred to 1 mL Terrific Broth (TB) in 24-well plates and incubated with shaking until the OD_600nm_ of the bacterial cultures reached approximately 0.6. Then, 100 µL of 1 mM isopropyl-β-d-thiogalactopyranoside (IPTG) was added to the TB to induce VHH antibody expression, and the culture was incubated at 37 °C overnight. After freezing and thawing three times, the supernatant was collected. Specific antibodies were identified by indirect ELISA. Rabbit anti-E tag antibody (Abcam, UK; ab3397) was used as the primary antibody, and goat anti-rabbit IgG H + L (HRP) (Abcam, UK; ab205718) as secondary. Bacterial fluids from positive clones (OD_450nm_ more than 3 times the control) were sent for sequencing and the sequence results were analyzed.

### Epitope analysis by additive ELISA

To determine whether Nbs recognize different epitopes on PEDV antigen, a summed ELISA analysis was performed to calculate the addition index (AI). Briefly, 96-well plates were coated with PEDV S1 protein (10 µg/mL) and incubated overnight at 4 °C. Addition of Nb alone or in combination that can saturate the coated antigen. After incubation and washing, the bound Nbs were detected by the addition of a rabbit anti-E tag antibody and goat anti-rabbit IgG H + L(HRP). PBS was used as a negative control. Competition for each Nb was determined by calculating the AI using the following equation: AI = [2A_1 + 2_ / (A_1_ + A_2_) − 1] × 100%, where A_1_ and A_2_ represent the OD_450nm_ values for each of two Nbs tested, and A_1 + 2_ represents the OD_450nm_ value when the two Nbs were mixed. If the AI is above 50%, then the two Nbs recognize different antigenic epitopes; if it is below 50%, then the two Nbs recognize the same antigenic epitope.

### Production of nanobody

Monovalent nanobody genes and biparatopic and bivalent nanobodies modified with Gly4Ser flexible linkers were individually cloned into the pET-21a expression vector. The vector was synthesized by Sangon Biotech (Shanghai) Co., Ltd. The recombinant plasmids were transformed into *E. coli* BL21(DE3) for nanobody expression. Cells were grown in LB at 200 rpm and 37 °C until the OD_600nm_ of the bacterial culture reached approximately 0.6. A variety of IPTG induction concentrations, temperatures and times were used to optimize the expression conditions. The bacterial cells were lysed by sonication and the nanobody protein was purified using a Ni-NTA resin column (CWBIO, China, CW0893S) according to the manufacturer’s instructions. Purified recombinant proteins were analyzed by 12% sodium dodecyl sulfate-polyacrylamide gel electrophoresis (SDS-PAGE). Subsequently, the eluted proteins were dialyzed for renaturation, quantified using a BCA Protein Assay Kit, and stored at -80 °C.

### Nanobody characterization

#### Specificity analysis

Common porcine pathogens, including PEDV, PRRSV, CSFV, PRV as well as PCV-2, were used to determine whether Nbs binds specifically to PEDV. Briefly, 10 µg/mL of virus was coated in an ELISA plate, the plate was blocked and Nbs was added. After incubation at 37 °C for 2 h, rabbit anti-His antibody and goat anti-rabbit IgG H + L(HRP) were added and the OD_450nm_ value was recorded after color development by TMB.

#### Stability analysis

The − 80 °C stored proteins were used as initial frozen Nbs, Nbs were collected and stored at 4 °C, and Nbs were aspirated from 4 °C EP tubes and stored at -80 °C weekly. After six weeks, the binding rate of Nbs to antigen was analysed by indirect ELISA. The Nbs were placed in a water bath at 30–90℃ for 1 h and in Tris-HCl at pH 3–11 for 1 h, respectively, and then the binding activity was detected by indirect ELISA. The results were expressed as relative activity (%) = (final Nb / initial Nb) × 100%.

#### Affinity analysis

PEDV virus was coated in ELISA plates at 10 µg/mL and incubated at 4 °C overnight. After three washes, the samples were blocked with 1% BSA. The Nb was uniformly diluted to an initial concentration of 1 mg/mL, and then the Nb was serially diluted 2-fold in the range of 1:2000-1:128000. The Nb dilution was added after three washes and incubated at 37 °C for 2 h, then rabbit anti-His tag antibody and goat anti-rabbit IgG H + L were added, and the OD_450nm_ value was recorded after TMB color development.

#### Cytotoxicity assay

The cytotoxic activity of Nbs on Vero and IPEC-J2 cells was determined using a Cell Counting Kit-8 (CCK-8; TargetMol, Shanghai, China). Briefly, approximately 1 × 10^5^ cells/well were seeded in a 96-well cell culture plate and incubated in a 5% CO_2_ humidified incubator at 37 °C. After confluent monolayers formed, the cells were washed with PBS and then incubated with different concentrations of Nbs in 3% FBS medium for 24 h. CCK-8 reagent (10 µL) was added to each well and incubated for 2 h at 37 °C. The OD value at 450 nm was measured using a microplate reader. The results are expressed as the percentage of the optical density of the treated cells to that of the untreated control cells, which was defined as 100% viability.

#### Neutralization test

Referring to the microplate neutralization assay for detecting nanobody neutralizing activity reported by Song et al. [48]. The purified Nb and combined cocktail were adjusted to 1 mg/mL and filtered through a 0.22-µm membrane. Subsequently, Nb was diluted 2-fold and incubated with an equal volume of 1000 TCID_50_/mL PEDV at 37 °C for 1 h. Then, the Nb-virus mixture was inoculated onto a confluent monolayer of Vero cells in 96-well plates and incubated at 37 °C for 2 h. Replacement of fresh DMEM containing 5 µg/mL TPCK-treated trypsin. After 36 h of cell culture, the cells were fixed in 4% paraformaldehyde, permeabilized with 0.5% Triton X-100, and blocked with 1% BSA to block nonspecific binding sites. After three more washes with PBS, rabbit anti-S1 sera (homemade, 1:1000 dilution) was incubated with cells as the first antibody. After a second wash, goat anti-rabbit IgG (H + L) was used as the secondary antibody, and the OD_450nm_ value was recorded after color development by TMB.

#### Inhibition of PEDV replication by Nbs

Vero cells were seeded into 96-well plates at a density of 1 × 10^6^ cells/mL for about 24 h, Nbs were diluted to different concentrations (5, 10, or 20 µM) with DMEM, and the cells were co-treated with 1000 TCID_50_/mL PEDV. After culture for 2 h at 37 °C with 5% CO_2_, the incubation mixture was discarded, the cells were washed three times with PBS, and fresh DMEM containing 5 µg/mL TPCK-treated trypsin was added. The culture was continued until the designated time points, and then the cell supernatant was collected for progeny virus titration. The cells were also harvested for quantitative reverse transcription PCR analyses.

Based on the previous experiments, we explored the inhibition linkage of viral replication by Nbs. A total of five treatments were performed before and after virus infection. The experiment included five different treatments: Treatment 1 involved pre-treatment of cells with Nbs for 1 h before virus infection; Treatment 2 consisted of co-incubation of cells with Nbs and viruses; Treatment 3 entailed pre-mixing of viruses and Nbs at 37 °C for 1 h, followed by treatment of cells; Treatment 4 involved incubation of viruses and cells at 4 °C for 2 h, followed by Nbs treatment; and finally, Treatment 5 consisted of performing Nbs treatment 2 h after viral infection had invaded the cells. Finally, the cell supernatant was collected for progeny virus titration and the cells were also harvested for quantitative reverse transcription PCR analyses.

#### Indirect immunofluorescence assay (IFA)

At indicated time points post viral infection, cells were fixed with 4% paraformaldehyde at room temperature (RT) for 15 min and then permeabilized with 0.5% Triton X-100 at RT for 5 min. After washing with PBS three times, the fixed cells were blocked by 1% BSA for 1 h at room temperature. After three more washes with PBS, Rabbit anti-His antibody at a dilution of 1:400 or rabbit anti-S1 sera at a 1:200 dilution was used as the primary antibody, and incubated with gentle shaking for 1 h at RT. The cells were then incubated with goat anti-rabbit IgG-FITC (Absin, Shanghai, China; abs2C023) at a 1:100 dilution as the secondary antibody for 1 h in the dark at RT. Nuclei were stained with DAPI (Solarbio, China) and all fluorescent images were acquired and analyzed using an inverted fluorescence microscope (Carl Zeiss AG, Germany).

#### Viral titration assay

To determine the titers of progeny virus in cell culture supernatants, Vero cells seeded in 96-well plates at a density of 1 × 10^5^ cells/well were cultured overnight at 37 °C with 5% CO_2_. Then, 100 µL/well of ten-fold-diluted (from 10^− 1^ to 10^− 10^) supernatant samples (material taken from the Neutralization test) with DMEM was added to single-layer Vero cells in 96-well plates for eight repeats of each dilution, and 100 µL/well DMEM was added as a control group and incubated at 37℃ in 5% CO_2_. After incubated for 2 h, the virus suspension was then aspirated and supplemented with fresh DMEM containing 5 µg/mL TPCK-treated trypsin and continued to culture. Observing cytopathic lesions daily, the 50% tissue culture infective dose (TCID_50_) was calculated using the Reed-Muench method.

#### Quantitative reverse transcription PCR (qRT-PCR)

The Vero cells were rinsed thrice with PBS, and total RNA was extracted utilizing the RNeasy^®^ Mini Kit following the manufacturer’s instructions. Subsequently, qRT-PCR was conducted using the HiScript^®^ II One Step qRT-PCR SYBR Green Kit (Vazyme, China; Q221) on an ABI QuantStudio 5 Real-Time PCR System (Thermo Fisher Scientific, USA). The cellular glyceraldehyde-3-phosphate dehydrogenase (GAPDH) gene was concurrently detected as an internal reference control for N expression normalization. We quantified the relative expression levels of N genes using the 2^−ΔΔCt^ method. We used this method to determine the change in expression of a target gene normalized to the endogenous control gene (GAPDH).

### Statistical analysis

All experiments were conducted independently at least three times to ensure reproducibility of results. Student’s *t*-test was used to determine statistical significance between groups, or one-way analysis of variance (ANOVA) when more than two groups were compared. A *p*-value of < 0.05 was considered statistically significant.

### Electronic supplementary material

Below is the link to the electronic supplementary material.

Supplementary Material 1

Supplementary Material 2

## Data Availability

The data and materials applied in supporting the findings in this study are available from the corresponding author upon request.
