# Supplementary material for: Monovalent, bivalent and biparatopic nanobodies targeting S1 protein of porcine epidemic diarrhea virus efficiently neutralized the virus infectivity
Source: BMC Vet Res. 2024 Jul 30;20:336. doi: 10.1186/s12917-024-04151-3 (PMC11290301; doi:10.1186/s12917-024-04151-3)
Supplement: Supplementary file 1 — Supplementary Material 1 [file 12917_2024_4151_MOESM1_ESM.docx]

| **Gene** | **Forward sequence** | **Reverse sequence** |
| --- | --- | --- |
| CALL | GTCCTGGCTGCTCTTCTACAAGG | GGTACGTGCTGTTGAACTGTTCC |
| VHH | CAGGTGCAGCTGCAGGAGTCTGGGGGAGR | CTAGTGCGGCCGCTGAGGAGACGGTGACCTGGGT |
| transformant | AATACGCAAACCGCCTCTCC | CTAGTGCGGCCGCTGAGGAGACGGTGACCTGGGT |
| pCANTAB-5E | CCATGATTACGCCAAGCTTTGGAGCC |  |
| PEDV N | ACTAATAAAGGGAATAAGGACCAG | GTTAGTGGGTTCAGTCTTTGC |
| hGAPDH | GATTCCACCCATGGCAAATTC | CTGGAAGATGGTGATGGGATT |
