# Supplementary material for: Monovalent, bivalent and biparatopic nanobodies targeting S1 protein of porcine epidemic diarrhea virus efficiently neutralized the virus infectivity
Source: BMC Vet Res. 2024 Jul 30;20:336. doi: 10.1186/s12917-024-04151-3 (PMC11290301; doi:10.1186/s12917-024-04151-3)
Supplement: Supplementary file 2 — Supplementary Material 2 [file 12917_2024_4151_MOESM2_ESM.docx]

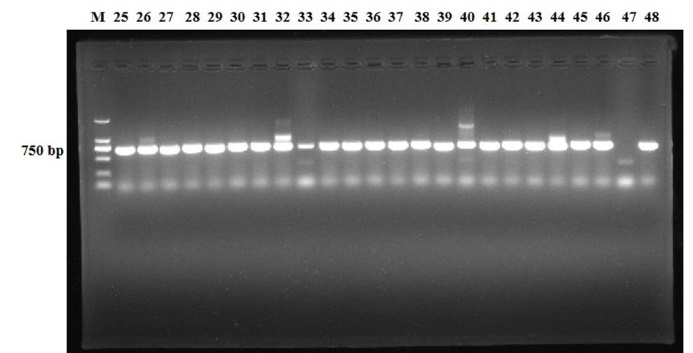

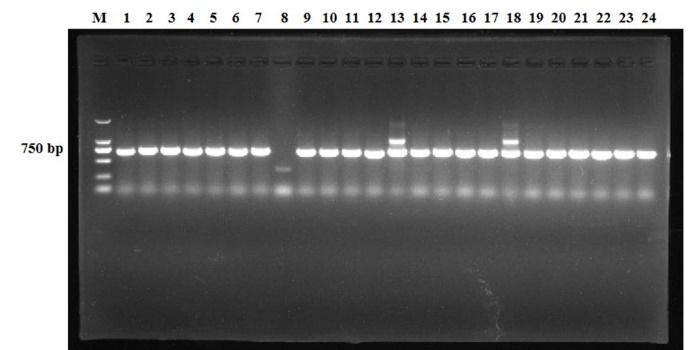

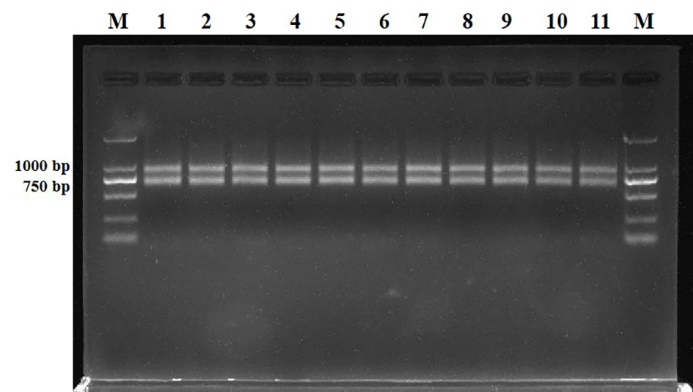


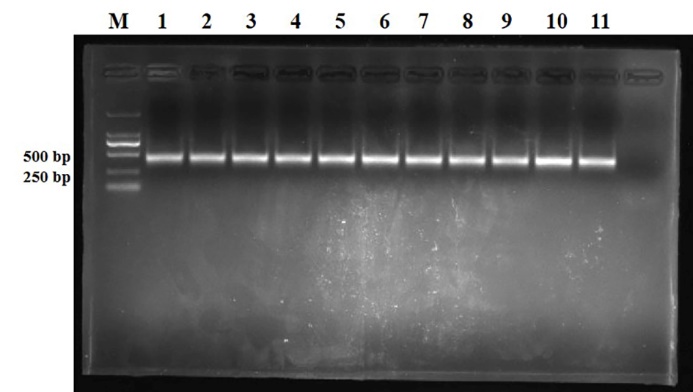


**Supplementary Figure 3. Evaluate the positive rate of the VHH library by colony PCR**

**Supplementary Figure 2. Second round of PCR amplification of the VHH gene**

**Supplementary Figure 1. First round of PCR amplification of the VHH gene**


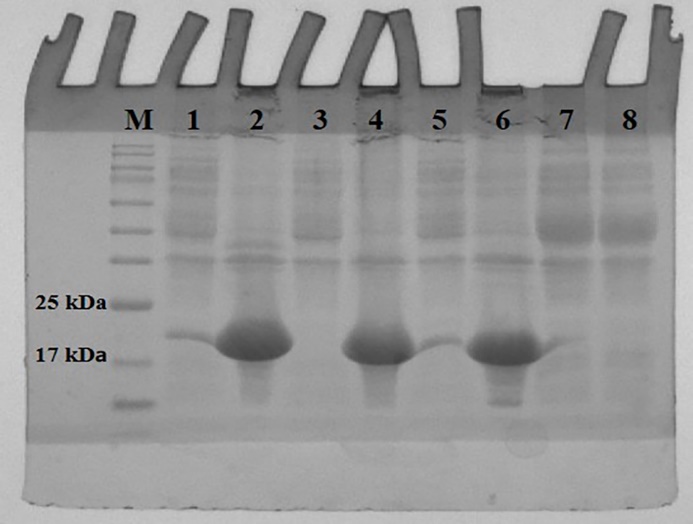


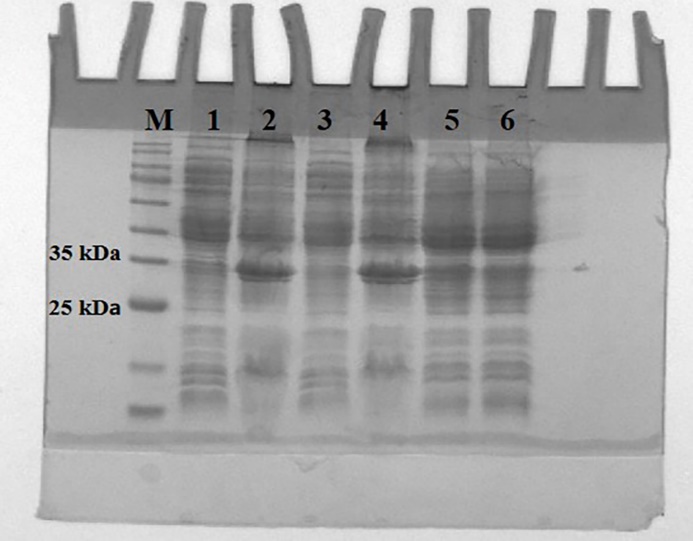

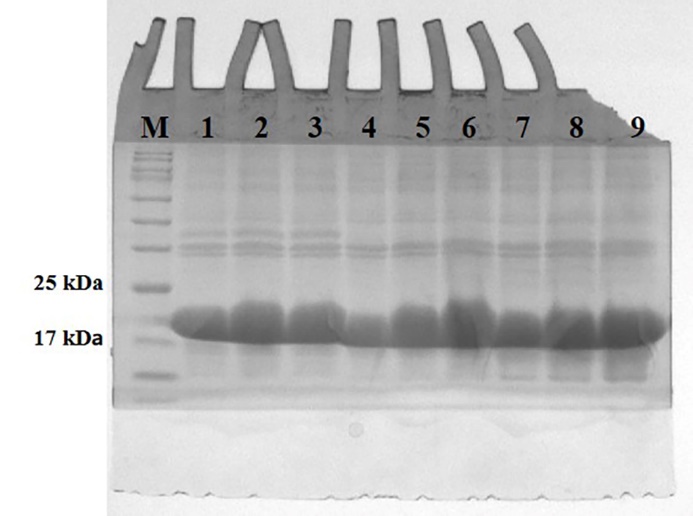


**Supplementary Figure 6. Optimization of concentration-inducing conditions for monovalent nanobody IPTG.** lane M: Solarbio 180 Marker; lanes 1-3: SF1; lanes 4-6: SF2; lanes 7-9: SF3; IPTG concentrations were 0.1 mM, 0.5 mM, and 1.0 mM in that order.

**Supplementary Figure 5. Solubility analysis of recombinant proteins with multivalent nanobodies.** lane M: Solarbio 180 Marker; lanes 1 and 3: supernatant of bacterial lysate; lanes 2 and 4: precipitate of bacterial lysate; 5: non-induced empty vector bacteria; 6: induced empty vector bacteria.

**Supplementary Figure 4. Solubility analysis of recombinant proteins with monovalent nanobodies.** lane M: Solarbio 180 Marker; lanes 1,3 and 5: supernatant of bacterial lysate; lanes 2, 4 and 6: precipitate of bacterial lysate; 7: non-induced empty vector bacteria; 8: induced empty vector bacteria.


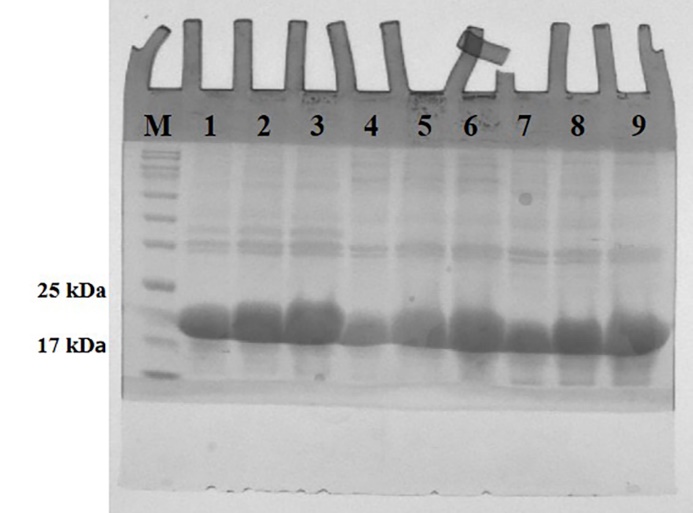


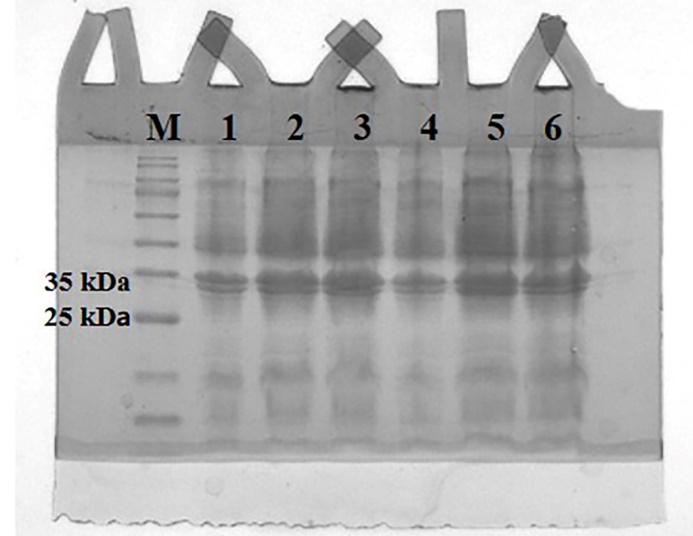

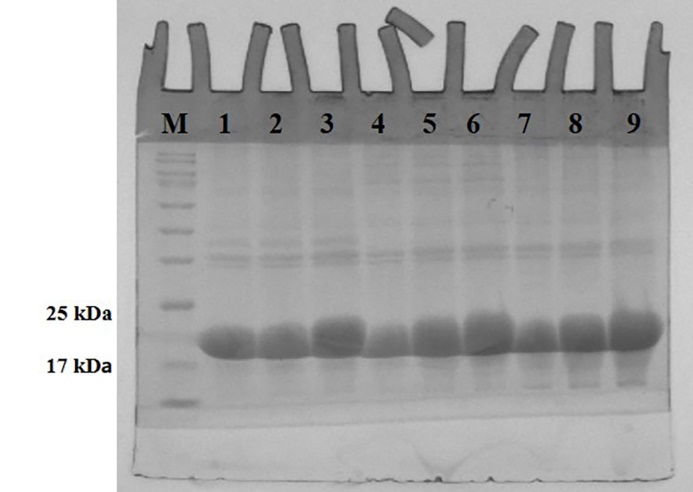


**Supplementary Figure 8. Optimization of temperature-induced conditions for IPTG with monovalent nanobodies.** lane M: Solarbio 180 Marker; lanes 1-3: SF1; lanes 4-6: SF2; lanes 7-9: SF3; the induction temperatures were 16°C, 28°C, and 37°C in that order.

**Supplementary Figure 9. Optimization of concentration-inducing conditions for multivalent nanobodies nanobody IPTG.** lane M: Solarbio 180 Marker; lanes 1-3: SFE lanes 4-6: SFB; IPTG concentrations were 0.1 mM, 0.5 mM, and 1.0 mM in that order.

**Supplementary Figure 7. Optimization of temporal induction conditions for IPTG with monovalent nanobodies.** lane M: Solarbio 180 Marker; lanes 1-3: SF1; lanes 4-6: SF2; lanes 7-9: SF3; the induction times were 4h, 8h and 12h in that order.


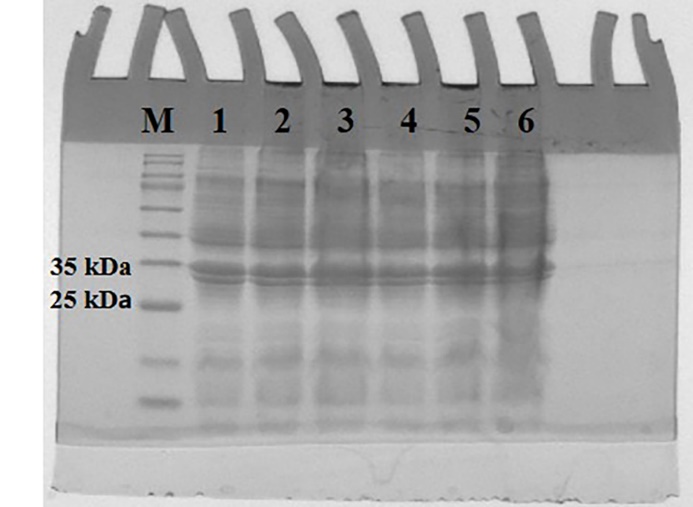


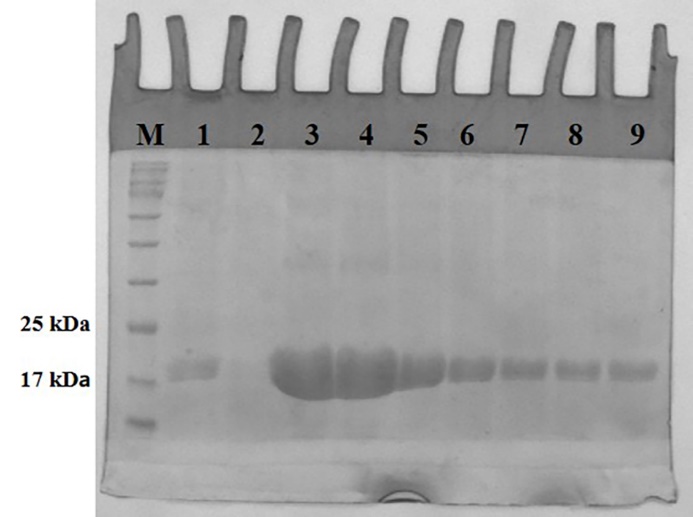

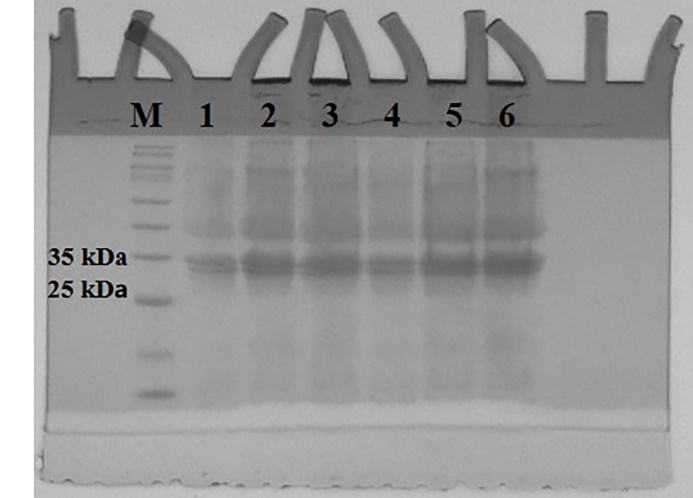


**Supplementary Figure 12. Identification of SF1 protein purification effect.** lane M: Solarbio 180 Marker; lane 1: flow-through solution; lane 2: wash solution; lanes 3-9: 3-9 mL elution solution.

**Supplementary Figure 11. Optimization of temperature-induced conditions for IPTG with multivalent nanobodies.** lane M: Solarbio 180 Marker; lanes 1-3: SF1; lanes 4-6: SF2; lanes 7-9: SF3; the induction temperatures were 16°C, 28°C, and 37°C in that order.

**Supplementary Figure 10. Optimization of temporal induction conditions for IPTG with multivalent nanobodies.** lane M: Solarbio 180 Marker; lanes 1-3: SF1; lanes 4-6: SF2; lanes 7-9: SF3; the induction times were 4h, 8h and 12h in that order.


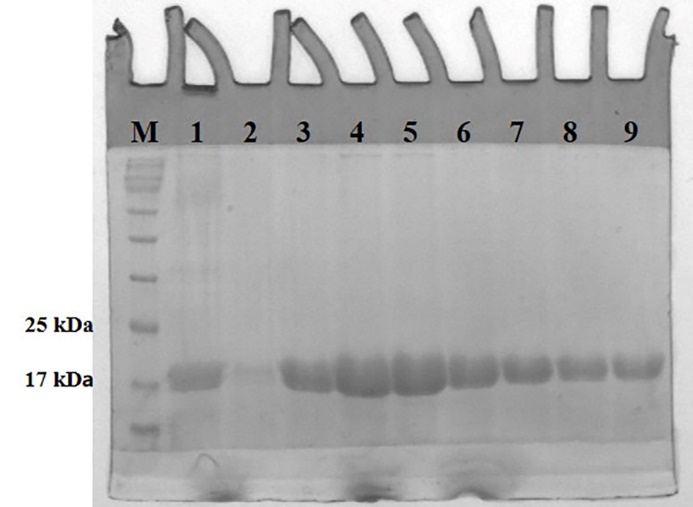


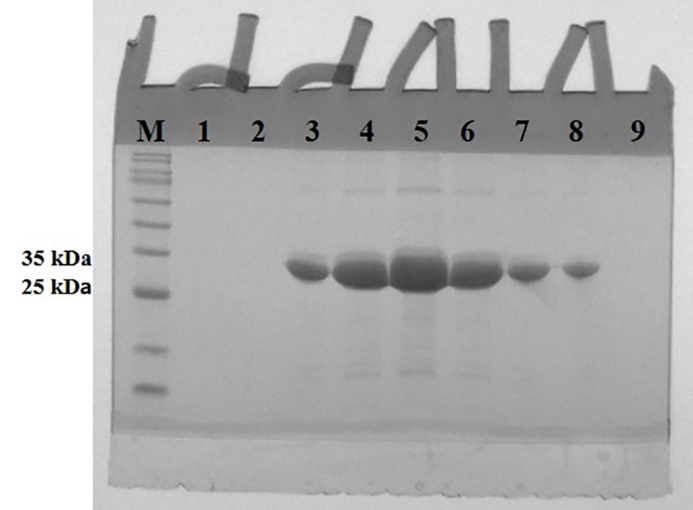

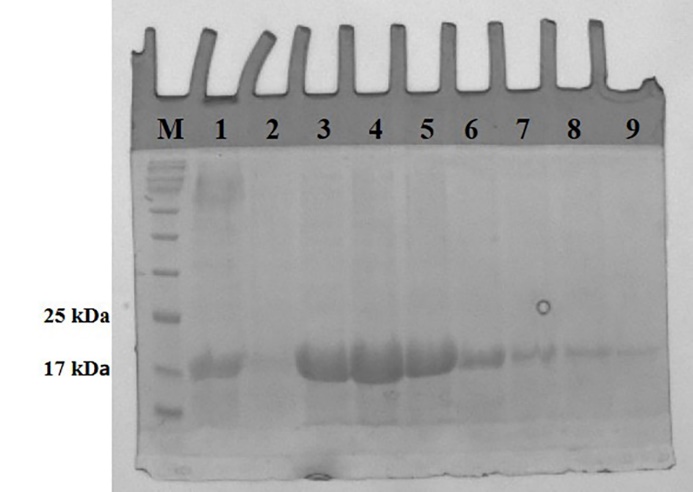


**Supplementary Figure 15. Identification of SFE protein purification effect.** lane M: Solarbio 180 Marker; lane 1: flow-through solution; lane 2: wash solution; lanes 3-9: 3-9 mL elution solution.

**Supplementary Figure 14. Identification of SF3 protein purification effect.** lane M: Solarbio 180 Marker; lane 1: flow-through solution; lane 2: wash solution; lanes 3-9: 3-9 mL elution solution.

**Supplementary Figure 13. Identification of SF2 protein purification effect.** lane M: Solarbio 180 Marker; lane 1: flow-through solution; lane 2: wash solution; lanes 3-9: 3-9 mL elution solution.


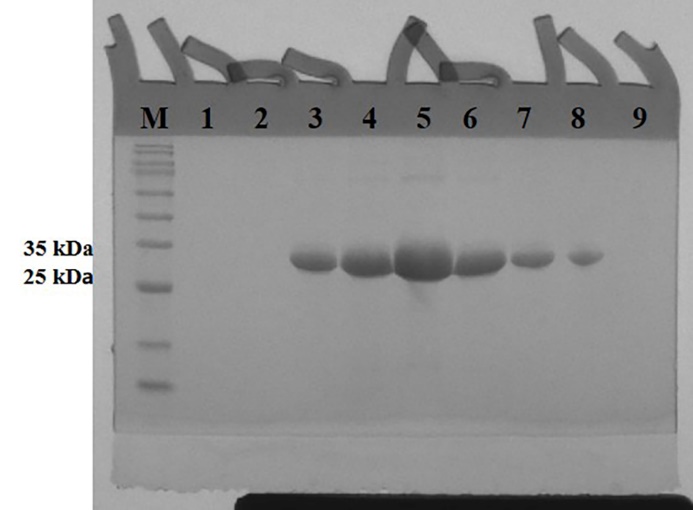


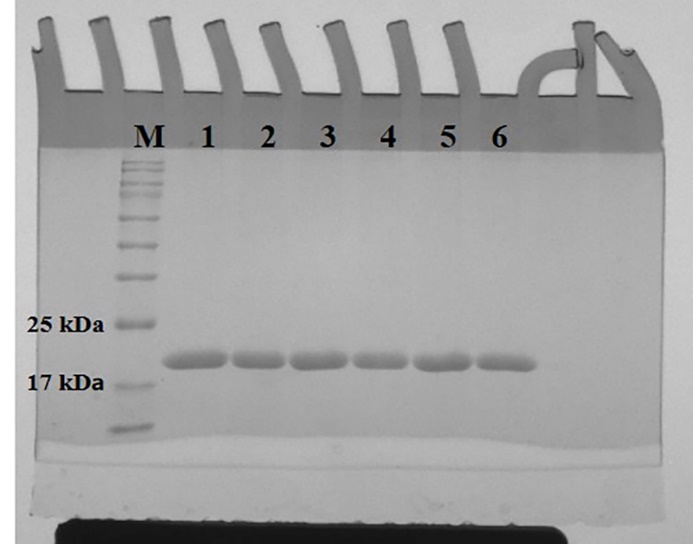

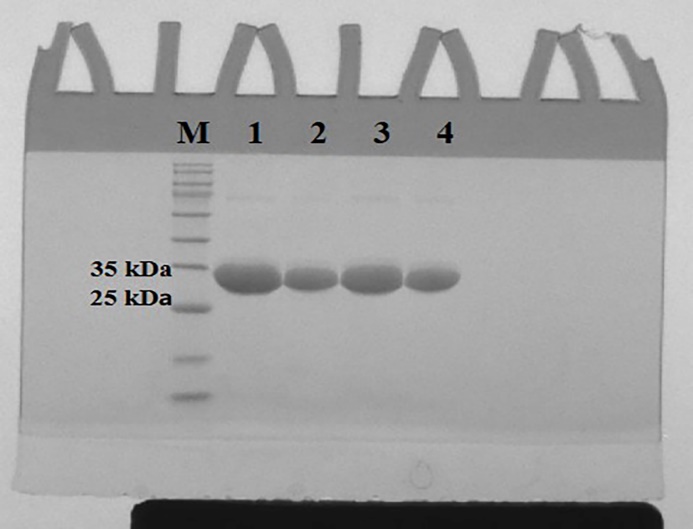


**Supplementary Figure 17. SDS-PAGE validation of monovalent nanobodies after reconstitution.** lane M: Solarbio 180 Marker; lane 1, 3 and 5 represent SF1, SF2 and SF3 before renaturing; lane 2, 4 and 6 represent SF1, SF2 and SF3 after renaturing.

**Supplementary Figure 18. SDS-PAGE validation of multivalent nanobodies after reconstitution.** lane M: Solarbio 180 Marker; lane 1 and 3 represent SF-E and SF-B before renaturing; lane 2 and 4 represent SF-E and SF-B after renaturing.

**Supplementary Figure 16. Identification of SFB protein purification effect.** lane M: Solarbio 180 Marker; lane 1: flow-through solution; lane 2: wash solution; lanes 3-9: 3-9 mL elution solution.


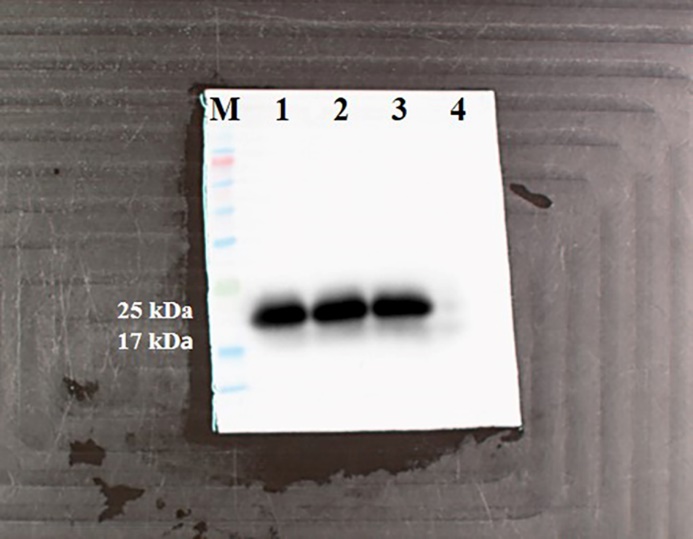


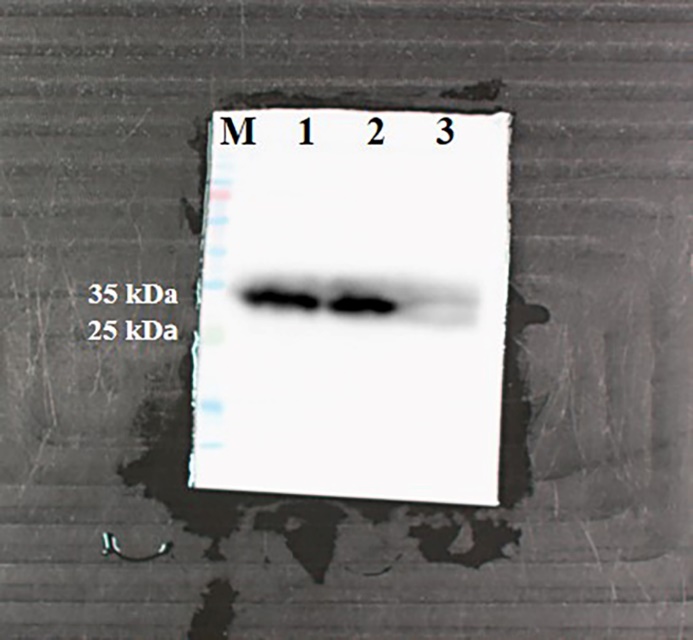


**Supplementary Figure 18. SDS-PAGE validation of multivalent nanobodies after reconstitution.** lane M: Solarbio 180 Marker; lane 1 and 2 represent SF-E, SF-B; lane 4: bacteria not induced by IPTG.

**Supplementary Figure 19. WB validation of monovalent nanobodies after reconstitution.** lane M: Solarbio 180 Marker; lane 1 to 3 represent SF1, SF2 and SF3; lane 4: bacteria not induced by IPTG.
